# Structure of Tris[2-(4-pyridyl)ethyl]phosphine, Tris[2-(2-pyridyl)ethyl]phosphine, and Their Chalcogenides in Solution: Dipole Moments, IR Spectroscopy, and DFT Study

**DOI:** 10.3390/molecules29010110

**Published:** 2023-12-23

**Authors:** Anastasiia A. Kuznetsova, Denis V. Chachkov, Natalia A. Belogorlova, Svetlana F. Malysheva, Yana A. Vereshchagina

**Affiliations:** 1 Department of Physical Chemistry, A.M. Butlerov Institute of Chemistry, Kazan Federal University, Kremlevskaya 18, 420008 Kazan, Russia; kuznetsovaanastan@gmail.com; 2 Kazan Department of Joint Supercomputer Center of Russian Academy of Sciences—Branch of Federal Scientific Center “Scientific Research Institute for System Analysis of the RAS”, Lobachevskogo 2/31, 420111 Kazan, Russia; de2005c@gmail.com; 3 A.E. Favorsky Irkutsk Institute of Chemistry, Siberian Branch of the Russian Academy of Sciences, Favorskogo 1, 664033 Irkutsk, Russia; belogorlova@irioch.irk.ru (N.A.B.); mal@irioch.irk.ru (S.F.M.)

**Keywords:** pyridylethylphosphine, phosphine chalcogenides, structure, dipole moments, conformational analysis, IR spectroscopy, DFT

## Abstract

Tris(hetaryl)substituted phosphines and their chalcogenides are promising polydentate ligands for the design of metal complexes. An experimental and theoretical conformational analysis of tris[2-(4-pyridyl)ethyl]phosphine, tris[2-(2-pyridyl)ethyl]phosphine, and their chalcogenides was carried out by the methods of dipole moments, IR spectroscopy and DFT B3PW91/6-311++G(df,p) calculations. In solution, these compounds exist as an equilibrium of mainly non-eclipsed (synclinal or antiperiplanar) forms with a predominance of a symmetrical conformer having a *gauche*-orientation of the C_sp3_–C_sp3_ bonds of pyridylethyl substituents relative to the P=X bond (X = lone pair, O, S, Se) and a *gauche*-orientation of the pyridyl rings relative to the zigzag ethylene bridges. Regardless of the presence and nature of the chalcogen atom (oxygen, sulfur, or selenium) in the studied molecules with many axes of internal rotation, steric factors—the different position of the nitrogen atoms in the pyridyl rings and the configuration of ethylene bridges—determine the realization and spatial structure of preferred conformers.

## 1. Introduction

Phosphines with pyridyl substituents at the phosphorus atom and their chalcogenide derivatives are known as polydentate ligands for the design of complex compounds due to the presence in their molecules of several coordination centers with different donor properties. Metal complexes based on pyridylphosphines ligands exhibit various types of luminescent properties [1,2,3,4,5,6,7,8,9,10,11,12], possess catalytic activity [13,14,15,16,17,18] (in particular, the ruthenium complex with tris(2-pyridyl)phosphine oxide is a pH-dependent electrocatalyst for water oxidation [19]), act as photoinducible CO-releasing molecules [20,21], and have magnetic properties [22,23,24]. Tris(3-pyridyl)phosphine has been used as a structure-forming moiety for the assembly of encapsulated transition metal catalysts based on porphyrins, which have the highest activity and selectivity in hydroformylation reactions [25,26]. The effect of conformational behavior on the catalytic performance of transition metals complexes was revealed in the case of the template ligand tris(3-pyridyl)phosphine [16,17]. Complexes of Ag(I) [27,28] and pyridyl-substituted phosphine sulfide and selenide [29] show biological activity. Phosphines with 2-pyridyl substituents are used as CO-prodrugs in the treatment of leukemia [21]. Complexes of tris[2-(2-pyridyl)ethyl]phosphine chalcogenides are promising as precursors for the production of metal phosphides and selenides nanoparticles [30], and a complex of the phosphine oxide is used as a flame retardant for polymeric materials [31]. Considerable attention is devoted to the study of the coordination and donor properties of such polydentate ligands [32,33,34,35,36].

Published research includes X-ray diffraction data on the structure of complexes involving tris(2-pyridyl)phosphine [5,6,7,8,9,10,11,19,33,35] and its chalcogenides [9,18,22,23,35] and tris[2-(2-pyridyl)ethyl]phosphine [3,36] and its chalcogenides [28,30] as ligands.

However, the information about the structure of pyridyl-substituted phosphines and their chalcogenides in the free state is scarce. The structures of tris(3-pyridyl)- and tris(4-pyridyl)phosphine chalcogenides were determined in the solid state [37]. The crystal structures of tris(2-pyridyl)phosphine and its chalcogenides were determined by X-ray diffraction [38,39,40]; in addition, we carried out the conformational analysis of these compounds in solution [41]. The structure of phosphines with pyridyl moieties separated by alkyl bridges from the phosphorus atom has been determined only for tris[2-(4-pyridyl)ethyl]phosphine oxide in the solid state [42], tris(2-pyridylmethyl)phosphine, and its derivatives [43,44].

The absence of a complete conformational picture for such hemilabile ligands complicates a comprehensive study of their reactivity, coordinating properties, and intra- and intermolecular interactions, which is necessary to establish the mechanisms of reactions involving these compounds.

Quantum chemistry methods are increasingly used to study of both the structure and properties of various ligands, including phosphorus-containing ligands and complexes based on them [7,17,32,36,45,46,47,48,49,50,51]. Using DFT modeling, tridentate *O*,*N*,*O*-donor cyclic dilactams were predicted to be much more selective and efficient extractants for the separation of lanthanides and actinides than open-structured pyridine-2,6-dicarboxamides, consistent with experimental data [45,46]. In addition, the radiolytic stability of conformationally flexible ligands—diglycolamides—was predicted [47]. The theoretical evaluation of a comparative affinity to actinide ions of bipyridine-dicarboxylic acid diamides was tested in terms of the DFT PBE0/TZ [49]. A novel approach for Am(III)/Eu(III) extraction efficiency modelling was presented in [48]: the solvent-corrected preorganization energy based on DFT calculations together with QTAIM analysis was proposed; this energy describes the behavior of the ligand and correlates with the extraction efficiency. A comparative analysis of various DFT methods and bases used for calculating the binding energies and selectivity of extractants and assessing a complex formation with f-elements was carried out in [51].

However, in the overwhelming majority of publications, studies using DFT, regardless of methods and bases, deal specifically with the structure and properties of complexes, not with free ligands.

Previously, we studied the structure in solution of a vast array of compounds of three- and four-coordinated phosphorus with multiple phosphorus–chalcogen bonds (chalcogen–oxygen, sulfur, selenium) and alkyl, aryl, and hetaryl substituents: tris(2-pyridyl)phosphine and its chalcogenides [41], *Se*-esters of diselenophosphinic acids [52], tri(1-naphthyl)phosphine, tri(2-naphthyl)phosphine, and their chalcogenides [53], and *N*,*N*-dibutylamide of dibutylphosphorylacetic acid [54]. A conformational analysis of these compounds was carried out using the methods of dipole moments, IR spectroscopy, and quantum chemistry B3PW91/6-311++G(df,p). The use of a complex of physical methods and quantum chemical calculations in the study of the relationship between the spatial structure and properties of molecules has a number of significant advantages, becoming a unique tool for establishing the structure of organoelement compounds in solutions. In all cases, the theoretical results were in good agreement with the obtained experimental data. The general structural regularities for these compounds indicate that their conformations fit into the overall conformational picture for compounds of tri- and tetra-coordinated phosphorus with alkyl and aryl substituents [55]. Namely, in solution, the studied compounds exist as a conformational equilibrium of several forms with a staggered *gauche*- and *trans*- or eclipsed *cis*-orientation of the substituents relative to the P=X bond (X = lone pair (LP), O, S, Se). The presence of eclipsed *cis*-conformations is explained by the formation of intramolecular H-contacts or the conjugation effect.

**Scheme 1:**
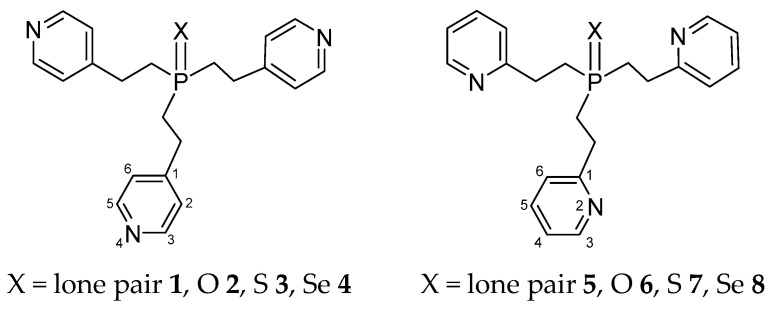
Structures of compounds **1**–**8**.

The purpose of the present research is to study the spatial structure of tris[2-(pyridin-4-yl)ethyl]phosphine **1**, tris[2-(pyridin-4-yl)ethyl]phosphine oxide **2**, tris[2-(pyridin-4-yl)ethyl]phosphine sulfide **3**, tris[2-(pyridin-4-yl)ethyl]phosphine selenide **4**, tris[2-(pyridin-2-yl)ethyl]phosphine **5**, tris[2-(pyridin-2-yl)ethyl]phosphine oxide **6**, tris[2-(pyridin-2-yl)ethyl]phosphine sulfide **7**, and tris[2-(pyridin-2-yl)ethyl]phosphine selenide **8** (Scheme 1) in solution by the method of dipole moments, IR spectroscopy, and DFT B3PW91/6-311++G(df,p) quantum chemical calculations. The identification of the features of the conformational behavior of these compounds depending on the position of the nitrogen atom in the pyridine rings and the arrangement of the ethylene bridges should facilitate research of their properties and reactivity, as well as explain the efficiency of a complex formation in solution.

## 2. Results and Discussion

### 2.1. Polarity of Compounds ***1***–***5***, ***7***, and ***8***

The method of dipole moments is a high-precision instrument for the conformational analysis of polar compounds in solution, and it is successfully used in combination with other physical methods and quantum chemistry to study the fine features of the spatial and electronic structure of organic and organoelement compounds [52,54,56].

For the first time, we have determined the polarities of compounds **1**–**5**, **7**, and **8**. The experimental values of the dipole moments were determined using the second Debye method based on the measurement of the dielectric permittivity of the dilute solutions of a polar substance in a nonpolar solvent [57]. The choice of solvent also depended on the solubility of the compounds: trichloromethane was used for **1**–**5**, **7**, and **8**, 1,4-dioxane—was used for **5**, and tetrachloromethane was used for **8**. The experimental dipole moments of **1**–**5**, **7**, and **8** are listed in Table 1.

The experimental values μ_exp_ were calculated by the Debye Equation (1) [57]:

(1) $$\mu =0.01283\sqrt{P_{or}T},$$

The orientation polarizabilities *P*_or_ were calculated by the Guggenheim–Smith Equation (2) [58,59]:

(2) $$P_{or}=\frac{M}{d}\left[\frac{3\alpha }{{\left(\varepsilon_{0}+2\right)}^{2}}-\frac{3\gamma }{{\left(n_{0}^{2}+2\right)}^{2}}\right]$$

where *M* is the molecular weight of a substance, *d* is the solvent density, α and γ are slopes of the *ε_i_*–*w_i_* and *n_i_*^2^–*w_i_* plots, and *ε_i_*, *n_i_*, and *w_i_* are the dielectric permittivity, refractive index, and weigh fraction of the solute of the *i*th solution, respectively. Equations for α and γ calculations (Guggenheim–Smith equation) as well as the ε*_i_*–*w_i_* and *n_i_*^2^–*w_i_* plots (Figures S1–S3, Table S1) are given in the Supplementary Materials.

In the calculations of dipole moments according to the vector-additive scheme, we used the theoretical geometry parameters and following moments of bonds and groups: *m*(P=>O) 3.40 D, calculated from μ_exp_ Et_3_P=O [56]; *m*(P=>S) 3.83 D, calculated from μ_exp_ Et_3_P=S [56]; m(P=>Se) 4.00 D, calculated from μ_exp_ Et_3_P=Se [56]; *m*(C_sp3_→P) 0.82 D [56]; *m*(C_sp3_→C_sp2_) 0.75 D [57]; *m*(pyridyl) 1.51 D, calculated from μ_exp_ pyridine [57]; and *m*(H→Csp2) 0.28 D [60].

The values of the dipole moments of compounds **2**–**4**, **7**, and **8** are quite high and are in good agreement with the known data on the polarities of the compounds of tetra-coordinated phosphorus with multiple phosphorus–chalcogen bonds and heteroaryl groups [56,57]. The high polarity values of tris[2-(4-pyridyl)ethyl]phosphine **1** and tris[2-(2-pyridyl)ethyl]phosphine **5** are due to the contribution of polar C–N bonds of the pyridine cycles and their positions relative to the P–C bonds. The same phenomenon was previously observed for tris(2-pyridyl)phosphine [41]. In addition, the high polarity values of compounds **1**–**5**, **7**, and **8** in trichloromethane solutions are apparently due to the formation of intermolecular interactions between the nitrogen atoms of the pyridine rings and the hydrogen atoms of the solvent molecules [61].

### 2.2. Experimental and Theoretical Conformational Analysis of Tris[2-(4-pyridyl)ethyl]phosphine ***1*** and Its Chalcogenides ***2***–***4***

The theoretical conformational analysis of phosphine **1** and its chalcogenides **2**–**4** was carried out using quantum chemical calculations by the DFT B3PW91/6-311++G(df,p) method. For **1**–**4**, several energetically preferred conformers were found, their relative energies and theoretical dipole moments were computed, and the dipole moments were calculated using the additive scheme (Table 2).

**Figure 1:**
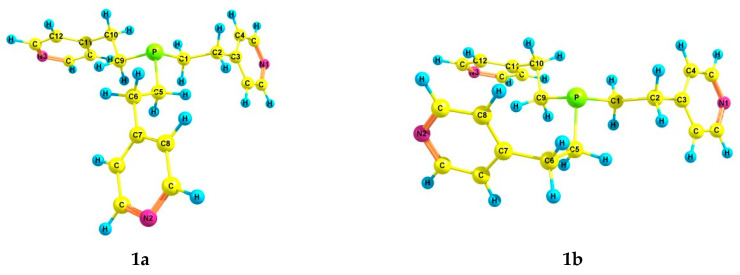

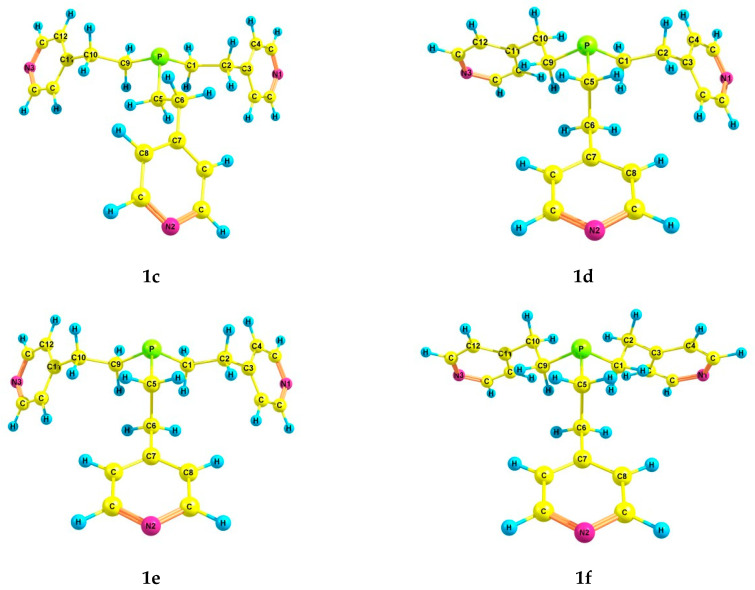
The preferred conformers of **1** according to DFT calculations.

According to the theoretical calculations for tris[2-(4-pyridyl)ethyl]phosphine **1**, six preferred conformers were found (Table 2 and Table 3, Figure 1). In conformers **1a**–**f**, the phosphorus atom is pyramidal, the C_sp3_–C_sp3_ bonds (ethylene bridges) are predominantly *gauche*-oriented relative to the axis P–lone electron pair (LP), and the pyridyl rings are *gauche*-oriented relative to the C_sp3_–C_sp3_ bonds. Conformer **1a** with zero relative energy has a symmetrical structure, and the ethylene bridges have a zigzag configuration. Conformer **1b** differs from **1a** in the arrangement of one of the substituents, in which the configuration of the ethylene bridge is pincer-like. Asymmetric conformer **1c**, as well as **1a**, has a zigzag configuration of the ethylene bridges. In **1d**, **1e**, and **1f**, one C_sp3_–C_sp3_ bond is *trans*-oriented relative to the axis P–LP, while all ethylene bridges have a zigzag configuration.

**Figure 2:**
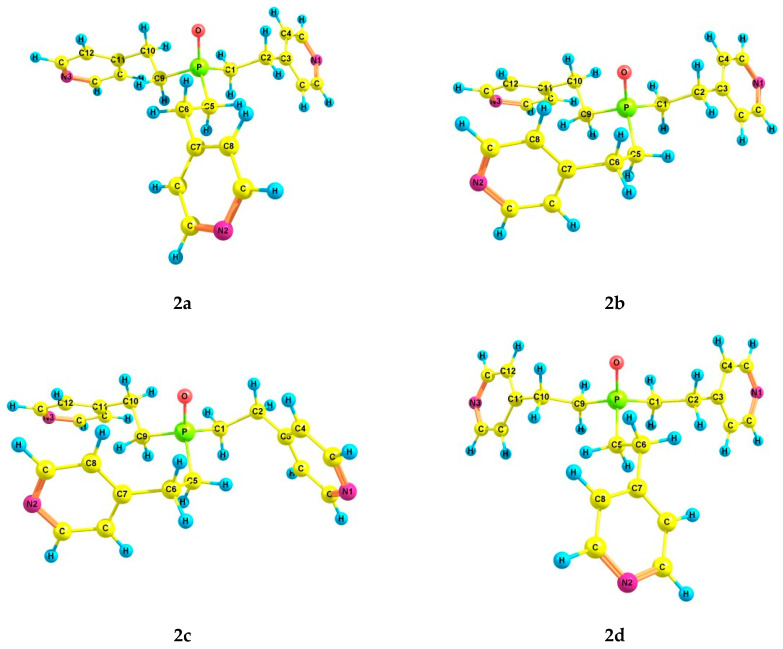

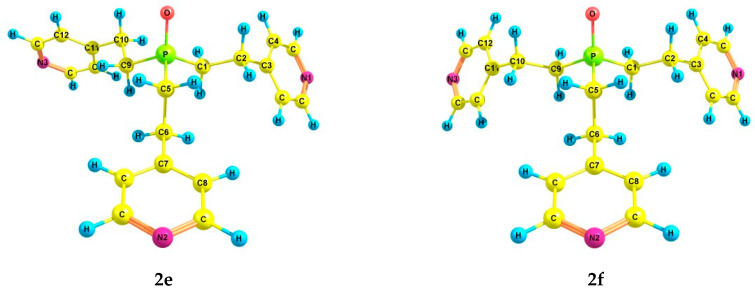
The preferred conformers of **2** according to DFT calculations.

An increase in the coordination of the phosphorus atom did not lead to an increase in the number of energetically preferred conformers. For phosphine oxide **2**, six conformers (**2a**–**f**) were also found (Table 2 and Table 3, Figure 2), in which the phosphorus atom is pyramidal, the C_sp3_–C_sp3_ bonds are mainly *gauche*-oriented relative to the phosphoryl group, and the pyridyl rings are *gauche*-oriented relative to the C_sp3_–C_sp3_ bonds. Symmetrical conformer **2a** with zero Δ*E* is characterized by a zigzag configuration of ethylene bridges. It should be noted that the structure of conformer **2a** corresponds to the structure of tris[2-(4-pyridyl)ethyl]phosphine oxide obtained by X-ray analysis [42]. In **2b**, one ethylene bridge has a pincer-like configuration, and in **2c**, two bridges are pincer-like. In conformer **2d**, one of the substituents is inverted compared to those in **2a**. Conformer **2e** differs from **2a** in the *trans*-orientation of one of the C_sp3_–C_sp3_ bonds. In conformer **2f**, one of the C_sp3_–C_sp3_ bonds is *trans*-orientated relative to the phosphoryl group, and the ethylene bridge is zigzag. Two other C_sp3_–C_sp3_ bonds are *gauche*-oriented relative to the P=O bond, and the ethylene bridges are pincer-like in these substituents.

For phosphine sulfide **3**, six energetically preferred conformers (**3a**–**f**) have the following common features: the pyramidal structure of the phosphorus atom, the predominant *gauche*-orientation of the C_sp3_–C_sp3_ bonds relative to the thiophosphoryl group, and the *gauche*-orientation of the pyridyl rings relative to the ethyl bridges (Table 2 and Table 3). As in the case of **1** and **2**, the symmetrical conformer **3a** with a zigzag configuration of the ethylene bridges has the minimum energy. A change in the configuration of one of the ethyl bridges to a pincer-like one leads to an increase in the energy of conformers **3b** and **3c**. Conformer **3d** differs from **3a** by a mirror inversion of one of the pyridylethyl substituents. In **3e** and **3f**, one C_sp3_–C_sp3_ bond is *trans*-oriented relative to the P=S group, and the bridges are zigzag.

According to the results of theoretical calculations, six conformers were found for phosphine selenide **4** as well (Table 2 and Table 3). In conformers **4a**–**f**, the phosphorus atom has a pyramidal structure, the C_sp3_–C_sp3_ bonds have a *gauche*-orientation relative to the P=Se group, and the pyridyl rings have a *gauche*-orientation relative to the C_sp3_–C_sp3_ bonds. The conformers **4a**–**f** differ in the configuration of the pyridylethyl substituents. In conformer **4a**, which has the minimum energy, the ethylene bridges are zigzag. A successive change in the configuration of the ethylene bridges to the pincer-like one leads to an increase in the energy of the conformers **4b** and **4c**. Conformer **4d** is similar to **4a**, with one of the pyridylethyl substituents mirror-inverted. In **4e** and **4f**, the *trans*-orientation of one of the C_sp3_–C_sp3_ bonds also leads to an increase in energy relative to **4a**.

**Figure 3:**
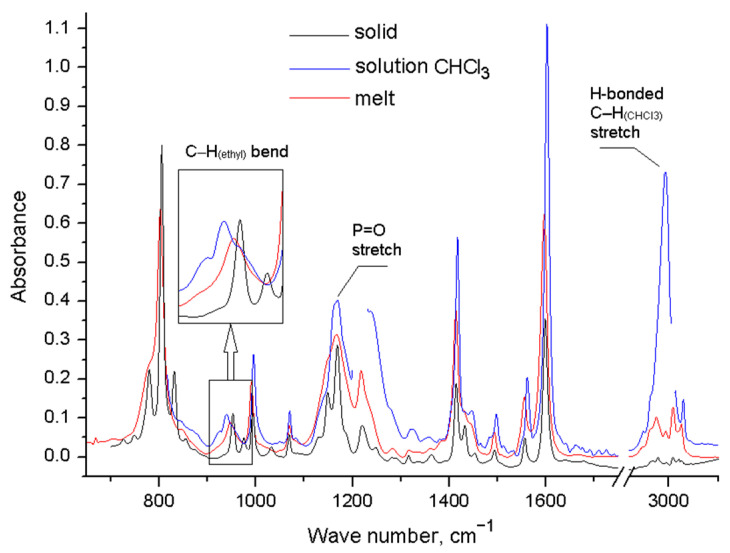
FT-IR spectra of compound **2** in different aggregate states.

We have registered the IR spectra of the compounds **2**–**4** in the solid state, in the melt, and in trichloromethane solution. In all spectra recorded for the solutions, there is a peak corresponding to the stretching vibrations of the bound C–H group of trichloromethane, which indicates the interaction of the substance with the solvent. The IR spectra of phosphine oxide **2** shows a change in the number of absorption bands in the melt and in solution (Figure 3, Table S1). The spectrum of solid **2** corresponds to the simulated spectrum of conformer **2a** with zero Δ*E*, the structure of which corresponds to the X-ray data [43]. In the region of bending vibrations of C–H bonds in ethylene bridges, one band at 953 cm^−1^ is observed. In the melt, this band is slightly shifted (ν = 948 cm^−1^), and a shoulder appears in the region of lower frequencies (about 920 cm^−1^); in this region, the vibrations of conformers with a different structure of substituents appear in the simulated spectra. The spectrum of the solution of **2** contains three bands—at 925 cm^−1^, 939 cm^−1^, and 952 cm^–1^—in this region, which indicates the appearance of other conformers.

In the case of compounds **3** and **4** (Figures S4 and S5, Table S2), a change in the number of bands in the spectra of solid and solution samples cannot be unambiguously stated. However, it should be noted that in the region of bending vibrations of C–H bonds in ethylene bridges, several bands or broadened bands with shoulders are observed both in the solid state and in solution, which indicates the presence of conformational heterogeneity.

Since, there are interactions between the solvent and molecules **2**–**4** according to the data of IR spectroscopy, we carried out theoretical calculations for the preferred conformers **1a**–**f**, **2a**–**f**, **3a**–**f**, and **4a**–**f** using the CPCM model and the cluster approach, taking into account the effect of the solvent (chloroform). The obtained energy characteristics and theoretical dipole moments are listed in Table 2. An analysis of the data calculated in the gas phase and in solution shows that taking into account the influence of the solvent led to an increase in the polarity of conformers, as well as to a significant increase in the relative energies and Gibbs energies for conformers with a pincer-like configuration of ethylene bridges. The percentage of preferred conformers for each of the compounds remained virtually unchanged.

A comparison of experimental (dipole moments, IR spectroscopy) and theoretical results indicates that, in solution, compounds **1**–**4** exist as a conformational equilibrium of several forms, with a predominance of a symmetrical conformer having a *gauche*-orientation of the C_sp3_–C_sp3_ bonds relative to the P=X group (X = LP, O, S, Se) and a *gauche*-orientation of the pyridyl rings relative to the zigzag ethylene bridges. In each substituent of the predominant conformers **1a**, **2a**, **3a**, and **4a**, the bonds X=P–C–C (X = LP, O, S, Se), P–C–C–C, and C–C–C=C have the conformation *A*, i.e., *synclinal*, *antiperiplanar*, and *synclinal* (the combination of dihedral angles α, δ, and η; β, ε, and θ; and γ, ζ, and ι, respectively, Table 3).

An analysis of the data in Table 2 shows that the use of the cluster approach did not meet our expectations: compared with the calculations in the gas phase and using the CPCM model, in many cases, there is an inconsistency in the values of the theoretical polarity of the preferred conformers. Therefore, we did not use the cluster approach in the theoretical calculations of compounds **5**–**8**.

### 2.3. Experimental and Theoretical Conformational Analysis of Tris[2-(2-pyridyl)ethyl]phosphine ***5*** and Its Chalcogenides ***6***–***8***

Theoretical calculations of tris[2-(2-pyridyl)ethyl]phosphine **5** and its chalcogenides **6**–**8** were carried out in the gas phase, as well as in solution using the CPCM model, which takes into account the effect of the solvent—1,4-dioxane, trichloromethane, or tetrachloromethane, depending on the conditions for determining the experimental polarity of each compound. According to the results of the theoretical calculations for compounds **5**–**8**, a large number of conformers with low relative energies were found (Table 4). Such conformational diversity is due to the presence of three mobile ethylene fragments in the molecules and the presence of the nitrogen atoms in the second position of the pyridine rings.

**Figure 4:**
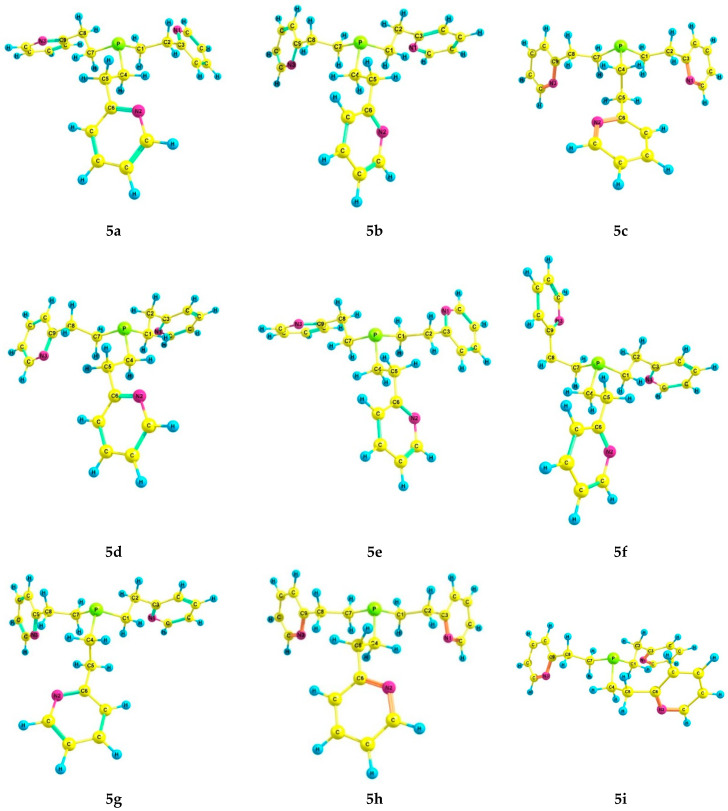

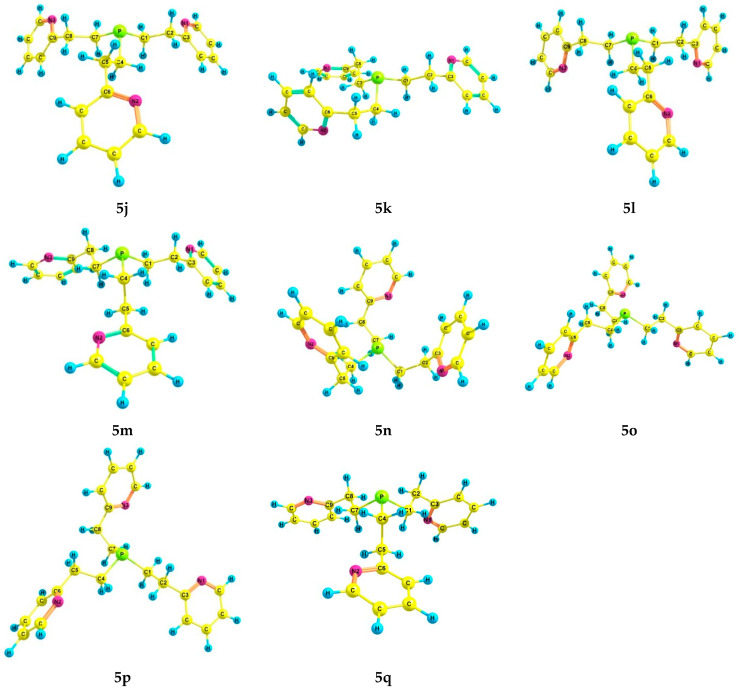
The preferred conformers of **5** according to DFT calculations.

For phosphine **5**, seventeen energetically preferred conformers were found (Figure 4); their characteristics are listed in Table 4. In each conformer (**5a**–**q**), the phosphorus atom has the pyramidal structure, and the pyridine rings have *gauche*-orientations relative to the ethylene bridges. The differences between the conformers lie in the orientation of the C_sp3_–C_sp3_ bonds relative to the P–LP axis and the configuration of the ethylene bridges (Table 5). In conformers **5a** and **5b**, which are symmetrical and have zero and close relative energies, the ethylene bridges have a zigzag configuration. A change in the configuration of one of the ethylene bridges in conformers to a pincer-like configuration leads to an increase in their relative energy.

**Figure 5:**
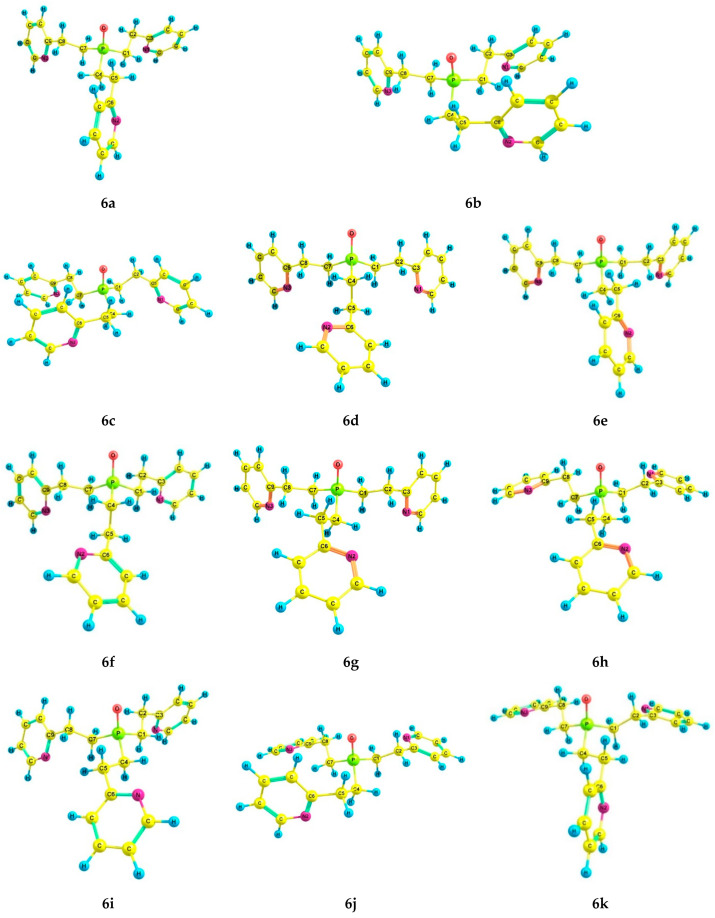
The preferred conformers of **6** according to DFT calculations.

With the introduction of the phosphoryl group, the number of preferred conformers of phosphine oxide **6** was reduced to eleven (Table 4, Figure 5). The conformers **6a**–**k** have the following common features: the pyramidal structure of the phosphorus atom and *gauche*-orientation of the pyridyl rings relative to the C_sp3_–C_sp3_ bonds in the pyridylethyl fragment (Table 5). In conformers **6a**–**c**, **6e**, and **6g**–**k**, the C_sp3_–C_sp3_ bonds of all three pyridylethyl substituents are *gauche*-oriented relative to the P=O group; in **6d** and **6f**, one of the ethylene bridges has a *trans*-orientation (Table 5), which leads to an increase in the energy of these conformers, as in the case of phosphine **5**. A successive change in the configuration of the ethylene bridges to a pincer-like configuration also leads to an increase in the relative energy in conformers **6b** and **6c**. In **6j**, one bridge is pincer-like, and the rest are zigzag.

For phosphine sulfide **7**, eleven energetically preferred conformers were also found (Table 4), in which the phosphorus atom is pyramidal; the pyridyl rings are *gauche*-oriented relative to the ethylene bridges (Table 5). The C_sp3_–C_sp3_ bonds are predominantly *gauche*-oriented relative to the P=S group in all conformers, except for **7c**, **7d,** and **7i**, where one of these bonds is *trans*-oriented (Table 5). In **7a**, **7c**, **7e**, **7f**, and **7h**–**k**, all ethylene bridges are zigzag. In **7b**, **7d**, and **7g**, the configuration of one ethylene bridge changes to be pincer-like.

For phosphine selenide **8**, twelve energetically preferred conformers were found (Table 4), in which the phosphorus atom is pyramidal and the pyridyl cycles are *gauche*-oriented relative to the ethylene bridges. The *gauche*-orientation of the C_sp3_–C_sp3_ bonds relative to the P=Se group is predominant, and a *trans*-orientation is observed only for one of the C_sp3_–C_sp3_ bonds in 8c, 8d, 8j, and **8k** (Table 5). All ethylene bridges are zigzag in **8a**, 8c, **8e**–**g**, and **8i**–**l**. A change in the zigzag configuration of one of the ethylene bridges to a pincer-like configuration leads to an increase in the energy of conformers **8b**, **8d**, and **8h**.

For all preferred conformers of compounds **5**, **7**, and **8**, the results of calculations using the CPCM model indicate an increase in the polarity of the conformers, expectedly more pronounced for trichloromethane than for1,4-dioxane and tetrachloromethane, which makes it possible to approach the values of the experimental polarity. Just as for compounds **1**–**4**, a significant increase in the relative energies and Gibbs energies is observed for conformers with a pincer-like configuration of ethylene bridges.

Because of the insufficient solubility of phosphine oxide **6** in nonpolar solvents, it is impossible to judge its conformation by the value of its experimental dipole moment, even when using the minimum concentration of solutions (0.001 mol/L).

To obtain additional information about the fine structural features of tris[2-(2-pyridyl)ethyl]-substituted derivatives, we have registered the IR spectra of **6**–**8** in the solid state, in the melt, and in solution (Table S3). For phosphine oxide **6**, new bands at 1165 cm^−1^, 1385 cm^−1^, and 1408 cm^−1^ appear in the spectrum of the melt compared to the solid sample; in the solid state, vibrations of the C–H bonds in ethyl fragments appear as a single band at 947 cm^−1^, and in the melt, they appear as a broadened band with shoulders in the region of higher and lower frequencies with a maximum at 939 cm^−1^. In the spectra of solutions of phosphine sulfide **7** and phosphine selenide **8** in trichloromethane, there is a band corresponding to stretching vibrations of the bound C–H bond in solvent and indicating the presence of intermolecular interactions. A comparison of the experimental and simulated IR spectral data indicates the presence of conformational heterogeneity for **6**–**8**.

The totality of the obtained experimental (dipole moments, IR spectroscopy) data and theoretical results allowed us to conclude that, in solution, compounds **5**–**8** exist as an equilibrium of several forms with a predominance of conformers characterized by a *gauche*-orientation of the C_sp3_–C_sp3_ bonds of pyridylethyl substituents relative to the P=X bond (X = LP, O, S, Se), a zigzag configuration of the ethylene bridges, and a *gauche*-orientation of the pyridyl rings relative to the ethylene bridges. As for compounds **1**–**4**, in each substituent of the predominant conformers **5a**, **6a**, **7a**, and **8a**, the bonds X=P–C–C (X = LP, O, S, Se), P–C–C–C, and C–C–C=N have the conformation *D* or *E* that is *synclinal*, *antiperiplanar*, and *synclinal* (the combination of dihedral angles κ, ν, and π; λ, ξ, and ρ; and µ, ο, and σ, respectively, Table 5).

An analysis of the data obtained in the gas phase and in solution shows that, as in the case of **1**–**4**, the use of the CPCM model in the calculations of compounds **5**, **7**, and **8** made it possible to increase the theoretical dipole moments of the preferred conformers independently of the solvent and, accordingly, to obtain better agreement with the experimental polarities of these compounds. At the same time, the relative energies and Gibbs energies, as well as the percentage of preferred conformers, practically did not change.

It should be emphasized that the introduction of ethylene bridges to the phosphorus atom in molecules **1**–**8**, as expected, led to a greater conformational diversity compared to tris(2-pyridyl)substituted phosphine and its chalcogenides, for which mainly a single conformer is present in solution [41].

## 3. Materials and Methods

### 3.1. Materials

Tris[2-(pyridine-4-yl)ethyl]phosphine **1** and tris[2-(pyridine-2-yl)ethyl]phosphine **5** were obtained as a result of the nucleophilic addition of phosphine PH_3_ to 2- and 4-vinylpyridines, proceeding to heating (65–70 °C) [62]. Tertiary phosphines **1** and **5** were used as building blocks for the synthesis of their chalcogenides; modified procedures for obtaining the phosphine chalcogenides **2**–**4** and **6**–**8** are presented in [62].

### 3.2. Dipole Moments

The experimental values of the dipole moments were determined according to the second Debye method [57]. The physical parameters of **1**–**5**, **7**, and **8** were measured from series of 4–6 solutions in a nonpolar solvent (1,4-dioxane, trichloromethane or tetrachloromethane) at 25 °C. The solvents were purified using the standard procedure. The dielectric permittivities of solutions of **1**–**5**, **7**, and **8** were determined on a BI-870 instrument (Brookhaven Instruments Corporation, New York, NY, USA); the accuracy is ±0.01. The refractive indices of the solutions were measured on an RA-500 refractometer (Kyoto Electronics, Kyoto, Japan); the accuracy is ±0.0001.

### 3.3. IR Spectroscopy

The infrared spectra of crystals were collected on an FTIR Bruker Vertex 70 spectrometer (Bruker, Ettlingen, Germany) with a single reflection, a germanium crystal ATR accessory (MIRacle, PIKE Technologies, Fitchburg, WI, USA) blown with dry air to remove atmospheric water vapor. The interferograms were registered in the region of 600–4000 cm^−1^ with a permission of 2 cm^−1^ and 128 scans and Fourier-transformed using a Blackman–Harris apodization function. The thin films of molten compounds were produced by the heating of the crystal between KBr plates. The temperature was measured by the PT100 sensor and was kept constant by the PID controller to provide a standard deviation smaller than 1 K. The crystallization of the films was visually monitored using crossed polarizers. The absence of the decomposition of the samples was proven by the identity of the original crystals spectra and the spectra of the solid phases after melting and subsequent crystallization. KBr cells were used with a spacer (0.2 mm) to achieve the best signal/noise ratio. For solutions, concentrations of samples were varied from 0.05 to 0.1 mol/L. Solvents—trichloromethane and tetrachloromethane—were purified by molecular sieves.

### 3.4. Quantum Chemical Calculations

Quantum chemical calculations with full geometry optimization were carried out in terms of the Density Functional Theory using the hybrid functional B3PW91 [63,64] and the 6-311++G(df,p) [65] extended basis set (calculations of the molecules in vacuum) by a Gaussian 09 software package [66]. We previously used the DFT method B3PW91/6-311++G(df,p) to study the polarity, structure, and reactivity of the compounds of tri- and tetra-coordinated phosphorus with aryl and hetaryl substituents and the P=O, P=S, and P=Se bonds [52,53,54]. The choice of this particular method is also based on the results of other studies—for example, [67]. Using the DFT B3PW91/6-311++G(df,p) method allows one to achieve good agreement between the values of theoretical and experimental dipole moments with an optimal calculation time.

Solvent effects were included in the framework of the Conductor-like Polarizable Continuum Model (CPCM) [68]. In all cases, the geometric parameters of the molecules were fully optimized. The accordance of the found stationary points to the energy minimums was proven by the calculation of the second derivatives of energy with respect to the atom coordinates. All structures identified as energy minima were characterized by Hessians containing only positive frequencies.

All possible conformations for compounds **1**–**8** were built by the successive rotation of the parts of the molecule relative to single bonds using the GaussView 6.0 [69] and Chemcraft Version 1.7 [70] imaging software. In all cases, we considered singlet molecules with a neutral charge. Conformations with overlapping atoms or with a too-close arrangement, which have no physical meaning, were rejected during the construction. At the first stage, the calculations were implemented using the small basis set 6-31G(d). According to the calculation results, degenerate structures and conformers with high values of relative energy (more than 14 kJ/mol) were discarded. Then, we performed the calculations in the extended basis set 6-311++G(df,p) and selected the preferred conformers with relative energies less than 7.6 kJ/mol while removing mirror isomers with the same dipole moments and relative energies. Next, refinement calculations were carried out for the preferred conformers in solution using the CPCM model, which takes into account the influence of the solvent and gives more accurate values of the dipole moments [52,54]. The percentage of conformers was calculated from the Gibbs free energies.

However, in the case of tetrachloromethane, the results of calculations using the CPCM model cannot be considered final, since according to the IR spectroscopy data, interactions of the compounds under consideration with trichloromethane were observed, and the continuum approach is not suitable for taking into account specific interactions. Therefore, we chose the cluster model with the explicit inclusion of solvent molecules for a more correct description of the structure and dipole moments of compounds **1**–**5**, **7**, and **8**. Calculations by the cluster method are laborious and time-consuming, but they take into account the specific solvation, which depends on the chemical nature of the solvent, and allow for the obtention of correct results in the case of the interaction between a solute and a solvent [71,72,73]. The cluster model involves the gradual introduction of solvent molecules around solute molecules until the primary solvation shell is saturated, resulting in the formation of a cluster due to specific solute–solvent interactions. Trichloromethane molecules were added sequentially until all sites in the molecule of the compound under study that could form H-contacts were saturated: the nitrogen atoms in pyridyl rings, the chalcogen atoms, and the orientation of the solvent molecule near the lone pair of the phosphorus atom was also possible. As each solvent molecule was added, the structure was optimized. Saturation was determined by a slight change in the energy of interaction between the molecules of the solute and the solvent when adding another molecule of trichloromethane, which characterized the beginning of the filling of the secondary solvation shell. In addition, the next solvent molecule was oriented near the already included solvent molecules, not the solute molecule. Thus, four-six trichloromethane molecules were taken into account in the primary solvation shell.

## 4. Conclusions

We have determined the polarities and carried out an experimental and theoretical conformational analysis of tris[2-(pyridin-4-yl)ethyl]phosphine, tris[2-(pyridin-4-yl)ethyl]phosphine oxide, tris[2-(pyridin-4-yl)ethyl]phosphine sulfide, tris[2-(pyridin-4-yl)ethyl]phosphine selenide, tris[2-(pyridin-2-yl)ethyl]phosphine, tris[2-(pyridin-2-yl)ethyl]phosphine oxide, tris[2-(pyridin-2-yl)ethyl]phosphine sulfide, and tris[2-(pyridin-2-yl)ethyl]phosphine selenide by the methods of dipole moments, IR spectroscopy, and DFT calculations.

The theoretical results are in good agreement with the experimental data. The combination of experimental methods, especially dipole moments, with DFT calculations, including using the CPSM model, made it possible to identify the general regularities of the conformational flexibility of the studied compounds with many axes of internal rotation in solution. Thus, the conformations of their molecules fit into the overall conformational picture for compounds of tri- and tetra-coordinated phosphorus with the phosphorus–chalcogen bonds and arylalkyl substituents at the phosphorus atom [52,53,54,55]. It was found that, in solution, compounds **1**–**8** exist as an equilibrium of mainly non-eclipsed (*synclinal* or *antiperiplanar*) forms, with a predominance of the symmetrical conformer having a *gauche*-orientation of the C_sp3_–C_sp3_ bonds of pyridylethyl substituents relative to the P=X bond (X = LP, O, S, Se) and a *gauche*-orientation of the pyridyl rings relative to the zigzag ethylene bridges.

On the other side, a comparison of the results obtained for tris[2-(4-pyridyl)ethyl]- and tris[2-(2-pyridyl)ethyl]-substituted phosphines and their chalcogenides indicates that a change in the position of the nitrogen atom in the pyridyl ring from the fourth to the second leads to the asymmetry of (2-pyridyl)ethyl substituents in molecules **5**–**8** and, accordingly, a significant increase in the number of preferred conformers in solution. Regardless of the presence and nature of the chalcogen atom in the molecules of phosphines with pyridylethyl substituents and their chalcogenides, it is the steric factors—the different position of the nitrogen atoms in the pyridine rings and the configuration of ethylene bridges—that determine the existence of preferred conformers and features of their spatial structure.

The results of this study have both theoretical and practical significance and will be useful for creating new complexes of tris[2-(4-pyridyl)ethyl]- and tris[2-(2-pyridyl)ethyl]-substituted phosphines and their chalcogenides with various metals. For a detailed study of the reactivity and complexing ability of these compounds, it is necessary to establish their structure in solution; it is the conformational flexibility of polydentate ligands that determines their properties and makes it possible to explain the efficiency of complexation in solution. Accordingly, conformational analysis of the molecules with many axes of internal rotation is undoubtedly a necessary step in predicting their various properties.

## Figures and Tables

**Table 1:** Coefficients in the calculation equations (α, γ), orientation polarizabilities (*P*_or_), and experimental dipole moments (μ) of compounds **1**–**5**, **7**, and **8**.

Compound	Solvent	α	γ	*P*_or_, cm^3^	µ, D
**1**	Trichloromethane	27.269	0.787	390.447	4.35
**2**	Trichloromethane	12.965	0.575	182.542	2.97
**3**	Trichloromethane	29.451	0.723	470.764	4.77
**4**	Trichloromethane	14.580	0.795	235.654	3.38
**5**	Trichloromethane	56.453	0.543	850.878	6.42
	1,4-Dioxane	4.348	0.429	224.203	3.29
**7**	Trichloromethane	41.956	0.606	681.889	5.74
**8**	Trichloromethane	36.222	0.646	654.633	5.63
	Tetrachloromethane	7.648	0.792	330.722	4.00

**Table 2:** Relative energies (Δ*E*, kJ/mol), Gibbs energies (Δ*E*, 298.15 K, kJ/mol), theoretical (μ_theor_) and calculated by additive scheme (μ_calc_) dipole moments (D), and percentage (*n*, %) of preferred conformers of **1**–**4** according to DFT B3PW91/6-311++G(df,p).

Conformer	μ_calc_	Gas Phase				Solution in CHCl_3_ (CPCM Model)				Solution in CHCl_3_ (Cluster Model)
		Δ*E*	Δ*G*	μ_theor_	*n*	Δ*E*	Δ*G*	μ_theor_	*n*	μ_theor_
**1a**	2.98	0.0	0.0	2.30	59.5	0.0	0.0	3.04	68.6	3.31
**1b**	5.00	3.3	7.0	4.02	3.5	3.3	4.6	5.16	1.8	7.69
**1c**	2.36	4.0	3.9	2.28	12.1	3.7	6.2	2.97	5.7	3.40
**1d**	2.30	4.2	5.0	1.83	8.0	5.0	9.0	2.34	10.7	2.63
**1e**	2.19	4.7	5.2	1.78	7.2	3.3	5.6	2.25	7.1	2.79
**1f**	2.50	7.5	4.6	1.91	9.3	6.6	6.1	2.45	6.0	2.91
**2a**	1.35	0.0	0.0	1.33	64.6	0.0	0.0	1.64	89.0	0.56
**2b**	5.47	1.1	4.6	4.27	10.2	4.7	12.4	5.49	0.6	6.50
**2c**	5.92	1.5	9.3	4.48	1.5	9.0	18.3	5.85	0.1	6.70
**2d**	1.76	4.2	4.7	1.55	9.7	4.1	9.5	1.82	1.9	1.81
**2e**	1.71	5.5	5.8	1.75	6.3	3.1	8.0	2.26	3.6	0.59
**2f**	1.87	5.6	5.5	1.87	7.2	2.7	7.2	2.44	4.9	1.52
**3a**	1.84	0.0	0.0	1.90	51.8	0.0	0.0	2.94	58.9	0.56
**3b**	5.46	25	4.8	4.28	7.6	4.6	6.9	5.89	3.7	6.84
**3c**	5.29	3.1	9.3	4.30	1.2	8.7	16.0	6.13	0.1	6.88
**3d**	2.89	4.2	1.9	2.12	23.7	4.4	4.2	3.02	10.6	0.82
**3e**	2.22	6.1	4.7	2.35	7.8	3.2	2.8	3.48	19.0	0.70
**3f**	2.10	6.2	4.7	2.25	7.7	3.5	5.2	3.40	7.3	0.49
**4a**	1.89	0.0	0.0	1.91	62.9	0.0	0.0	3.05	41.2	1.00
**4b**	5.48	2.6	6.6	4.27	4.4	4.6	6.0	5.96	3.6	6.96
**4c**	5.62	3.6	10.9	4.32	0.8	9.1	14.5	6.17	0.1	7.00
**4d**	2.35	3.9	2.6	2.15	21.8	3.7	0.2	3.13	37.6	0.98
**4e**	2.34	6.3	6.2	2.45	5.2	3.3	3.3	3.69	10.9	0.93
**4f**	2.22	6.4	6.3	2.35	4.9	3.7	4.6	3.63	6.5	0.22

**Table 3:** Selected dihedral angles (degrees) for preferred conformers **1**–**4** according to DFT B3PW91/6-311++G(df,p)+CPCM (solvent CHCl_3_): α X=P–C1–C2, β X=P–C5–C6, γ X=P–C9–C10, δ P–C1–C2–C3, ε P–C5–C6–C7, ζ P–C9–C10–C11, η C1–C2–C3=C4_,_ θ C5–C6–C7=C8, ι C9–C10–C11=C12.

	Conformation ^1^	α	β	γ	δ	ε	ζ	η	Θ	ι
**1a**	*AAA*	-	-	-	−178	−178	−178	89	89	89
**1b**	*ABA*	-	-	-	−179	66	−179	88	−105	89
**1c**	AA*A*	-	-	-	−175	178	179	89	−86	−89
**1d**	*ACA*	-	-	-	−175	−179	−178	87	−88	89
**1e**	AC*A*	-	-	-	−175	180	175	88	−89	−88
**1f**	A*CA	-	-	-	175	180	−175	−88	−89	88
**2a**	*AAA*	49	50	50	−176	−176	−176	88	89	88
**2b**	*ABA*	46	37	49	−177	73	−178	88	−114	89
**2c**	*BBA*	36	37	47	74	74	−179	−111	−114	89
**2d**	AA*A*	58	−34	−48	−173	174	178	88	−87	−89
**2e**	*ACA*	54	179	49	−175	−179	−176	88	−90	88
**2f**	AC*A*	56	180	−56	−174	180	174	87	−89	−87
**3a**	*AAA*	57	57	57	−174	−174	−174	84	85	85
**3b**	*ABA*	51	42	53	−176	73	−177	83	−116	86
**3c**	*BBA*	40	41	49	74	74	−178	−114	−116	86
**3d**	AA*A*	39	−59	−59	−174	171	174	83	−84	−85
**3e**	AC*A*	58	180	−58	−174	180	174	84	−89	−84
**3f**	*ACA*	58	180	54	−174	180	−174	85	−91	84
**4a**	*AAA*	56	56	56	−175	−175	−175	85	85	85
**4b**	*ABA*	51	43	53	−176	74	−177	86	−116	86
**4c**	*BBA*	42	42	50	75	74	−178	−115	−117	87
**4d**	AA*A*	56	−42	−52	−172	173	176	85	−84	−86
**4e**	AC*A*	58	180	−58	−174	180	174	84	−89	−84
**4f**	*ACA*	58	180	53	−175	180	−175	85	−90	84

^1^ Conformations of the substituents (the combination of dihedral angles α, δ, η; β, ε, θ or γ, ζ, ι): *A*—(*sc*,-*ap*,*sc*), *B*—(*sc*,*sc*,-*ac*), *C*—(*ap*,-*ap*,-*sc*); *sc—synclinal*, *ap—antiperiplanar*, *ac—anticlinal*.* Mirror conformation.

**Table 4:** Relative energies (Δ*E*, kJ/mol), Gibbs energies (Δ*E*, 298.15 K, kJ/mol), theoretical (μ_theor_) and calculated by additive scheme (μ_calc_) dipole moments (D), and percentage (*n*, %) of preferred conformers of **5**–**8** according to DFT B3PW91/6-311++G(df,p).

Conformer	μ_calc_	Gas Phase				Solution (CPCM Model)			
		Δ*E*	Δ*G*	μ_theor_	*n*	Δ*E*	Δ*G*	μ_theor_	*n*
**5a**	2.73	0.0	0.0	4.29	25.9	0.0 *, 0.0 **	0.0 *, 0.0 **	5.70 *, 6.69 **	24.6 *, 19.3 **
**5b**	1.52	1.6	0.1	0.93	25.0	0.8 *, 0.6 **	0.8 *, 0.2 **	1.31 *, 1.54 **	17.8 *, 17.6 **
**5c**	1.70	2.7	4.3	2.23	4.6	2.7 *, 2.9 **	3.5 *, 3.1 **	2.93 *, 3.38 **	5.9 *, 5.5 **
**5d**	1.09	3.0	3.5	1.95	6.2	2.9 *, 3.2 **	3.5 *, 2.7 **	2.28 *, 2.52 **	6.0 *, 6.4 **
**5e**	1.96	3.3	4.2	3.36	4.7	3.5 *, 3.7 **	3.2 *, 2.5 **	4.22 *, 4.77 **	6.7 *, 7.0 **
**5f**	2.09	4.1	4.0	3.02	5.0	3.2 *, 2.9 **	3.4 *, 2.2 **	3.84 *, 4.29 **	6.3 *, 8.0 **
**5g**	1.57	4.4	5.4	1.93	2.9	4.0 *, 3.7 **	4.9 *, 3.6 **	2.77 *, 3.31 **	3.4 *, 4.6 **
**5h**	1.89	4.5	3.5	2.47	6.4	4.0 *, 3.9 **	2.9 *, 2.6 **	3.28 *, 3.78 **	7.5 *, 6.8 **
**5i**	1.89	4.7	5.6	2.97	2.7	4.5 *, 4.6 **	5.4 *, 5.3 **	3.73 *, 4.20 **	2.8 *, 2.2 **
**5j**	3.55	4.9	5.0	5.02	3.4	4.3 *, 4.0 **	4.7 *, 2.7 **	6.45 *, 7.19 **	3.7 *, 6.5 **
**5k**	1.97	4.9	6.3	2.17	2.0	4.7 *, 4.7 **	6.4 *, 6.5 **	2.82 *, 3.24	1.9 *, 1.4 **
**5l**	1.88	5.5	5.1	1.82	3.3	4.8 *, 4.5 **	4.5 *, 3.4 **	2.67 *, 3.22 **	3.9 *, 4.8 **
**5m**	3.53	5.5	5.7	4.91	2.6	4.4 *, 3.8 **	4.4 *, 3.7 **	6.19 *, 6.99 **	4.1 *, 4.4 **
**5n**	0.66	5.5	8.6	3.01	0.8	5.8 *, 6.2 **	8.3 *, 9.0 **	3.33 *, 3.44 **	0.9 *, 0.5 **
**5o**	2.47	6.4	6.3	3.45	2.0	6.2 *, 6.2 **	7.6 *, 7.5 **	4.51 *, 5.11 **	1.1 *, 0.9 **
**5p**	2.98	7.3	7.3	2.86	1.3	6.8 *, 6.5 **	6.8 *, 6.3 **	3.79 *, 4.50 **	1.6 *, 1.5 **
**5q**	3.09	7.6	7.6	4.31	1.2	7.1 *, 6.8 **	6.5 *, 5.1 **	5.20 *, 5.71 **	1.8 *, 2.4 **
**6a**	1.20	0.0	0.0	0.87	46.0	-	-	-	-
**6b**	1.48	0.8	3.6	2.52	10.7	-	-	-	-
**6c**	1.40	1.4	6.9	2.64	2.8	-	-	-	-
**6d**	2.98	2.7	4.9	3.39	6.4	-	-	-	-
**6e**	1.44	4.3	5.2	1.42	5.7	-	-	-	-
**6f**	2.99	4.5	6.1	3.33	3.9	-	-	-	-
**6g**	2.55	4.6	5.4	2.57	5.1	-	-	-	-
**6h**	4.79	4.8	4.7	5.14	6.8	-	-	-	-
**6i**	3.16	5.3	6.1	3.23	3.9	-	-	-	-
**6j**	4.08	5.3	7.2	3.80	2.5	-	-	-	-
**6k**	3.61	6.3	6.9	3.74	2.9	-	-	-	-
**7a**	1.77	0.0	0.0	1.44	33.6	0.0 *, 0.0 **	0.0 *, 0.0 **	1.80 *, 2.03 **	42.6 *, 39.3 **
**7b**	2.17	0.0	2.8	2.72	11.0	1.9 *, 2.5 **	3.5 *, 5.2 **	3.38 *, 3.82 **	10.3 *, 4.9 **
**7c**	3.57	2.1	4.2	4.18	6.1	2.1 *, 1.6 **	3.98, 2.9 **	5.00 *, 5.53 **	8.8 *, 12.0 **
**7d**	3.34	3.0	8.5	4.94	1.1	4.3 *, 4.4 **	8.1 *, 8.5 **	5.97 *, 6.60 **	1.6 *, 1.3 **
**7e**	4.25	3.1	3.2	4.72	9.4	5.9 *, 4.9 **	6.5 *, 6.4 **	6.13 *, 7.41 **	3.1 *, 3.0 **
**7f**	1.76	3.9	2.5	1.93	12.3	4.6 *, 4.4 **	4.4 *, 4.3 **	2.52 *, 3.00 **	7.2 *, 6.8 **
**7g**	4.35	4.1	6.4	4.39	2.5	5.0 *, 4.1 **	7.3 *, 7.8 **	6.35 *, 7.92 **	2.3 *, 1.7 **
**7h**	5.21	4.1	3.3	5.62	9.0	4.4 *, 2.3 **	4.4 *, 3.7 **	8.49 *, 11.78 **	7.2 *, 8.9 **
**7i**	3.37	4.3	5.8	4.06	3.3	3.7 *, 2.8 **	4.9 *, 3.8 **	4.95 *, 5.54 **	6.0 *, 8.6 **
**7j**	2.90	4.3	4.2	3.14	6.2	4.7 *, 4.2 **	4.6 *, 4.0 **	4.42 *, 5.28 **	6.8 *, 7.8 **
**8a**	2.03	0.0	0.0	1.78	46.3	0.0 ***, 0.0 **	0.0 ***, 0.0 **	2.21 ***, 2.52 **	47.1 ***, 34.5 **
**8b**	2.31	0.1	4.1	2.88	9.0	1.6 ***, 2.2 **	4.9 ***, 4.7 **	3.60 ***, 4.03 **	6.4 ***, 5.1 **
**8c**	3.55	2.8	5.9	4.40	4.4	2.2 ***, 1.9 **	4.7 ***, 4.4 **	5.29 ***, 5.88 **	7.2 ***, 5.9 **
**8d**	3.48	3.6	8.8	5.14	1.3	4.3 ***, 4.6 **	8.7 ***, 7.7 **	6.21 ***, 6.21 **	1.4 ***, 1.6 **
**8e**	1.89	4.3	4.8	2.11	6.7	4.2 ***, 4.2 **	5.0 ***, 4.2 **	2.78 ***, 3.29 **	6.3 ***, 6.4 **
**8f**	5.09	4.4	3.6	5.64	10.7	4.1 ***, 2.4 **	4.2 ***, 3.5 **	8.59 ***, 10.87 **	8.6 ***, 8.3 **
**8g**	3.05	4.4	5.1	3.32	5.9	4.5 ***, 4.0 **	5.2 ***, 4.0 **	4.68 ***, 5.60 **	5.7 ***, 6.8 **
**8h**	4.43	4.6	7.4	4.51	2.4	5.0 ***, 4.2 **	8.7 ***, 6.8 **	6.66 ***, 8.12 **	1.4 ***, 2.2 **
**8i**	2.91	4.7	5.0	3.09	6.2	4.6 ***, 4.2 **	5.4 ***, 3.4 **	4.25 ***, 4.03 **	5.2 ***, 8.9 **
**8j**	3.54	5.0	7.2	4.31	2.5	3.9 ***, 2.8 **	5.2 ***, 2.4 **	5.28 ***, 5.92 **	5.7 ***, 12.9 **
**8k**	4.08	6.1	6.8	4.46	2.9	5.7 ***, 4.6 **	7.0 ***, 4.9 **	6.37 ***, 7.72 **	2.8 ***, 4.9 **
**8l**	5.15	7.0	8.3	6.22	1.6	5.1 ***, 5.4 **	7.6 ***, 6.6 **	8.00 ***, 11.50 **	2.2 ***, 2.4 **

* 1,4-dioxane, ** trichloromethane, *** tetrachloromethane.

**Table 5:** Selected dihedral angles (degrees) for the preferred conformers (**5**–**8**) according to DFT B3PW91/6-311++G(df,p)+CPCM: κ X=P–C1–C2, λ X=P–C4–C5, µ X=P–C7–C8, ν P–C1–C2–C3, ξ P–C4–C5–C6, ο P–C7–C8–C9, π C1–C2–C3=N1, ρ C4–C5–C6=N2, and σ C7–C8–C9=N3.

	Conformation ^1^	κ	λ	µ	ν	ξ	ο	π	Ρ	σ
**5a**	*DDD*				−177	−176	−176	71	72	72
**5b**	*EEE*				−179	−179	−179	79	79	79
**5c**	E*GE				−178	−179	177	−78	74	75
**5d**	*EDE*				−179	−173	−179	78	72	78
**5e**	*DED*				−173	180	−175	71	78	72
**5f**	*EEH*				180	180	68	77	79	59
**5g**	*EGE*				179	−179	177	77	74	76
**5h**	E*DE				179	−176	176	−77	74	80
**5i**	EF*E				−179	−70	−178	78	−68	79
**5j**	DDD*				−178	−176	173	70	66	−72
**5k**	*DFD*				−177	71	−178	73	68	77
**5l**	E*EE				−176	180	−179	−77	79	78
**5m**	*DGD*				−174	−178	−177	71	77	73
**5n**	*HHH*				69	69	69	61	60	60
**5o**	EE*F				180	−177	70	76	−79	60
**5p**	*DDH*				−175	−173	69	73	72	60
**5q**	*EGD*				180	−179	−173	77	76	72
**6a**	*EEE*	−46	−46	−46	179	179	179	67	66	66
**6b**	EF*E	−48	−32	−45	180	−74	−179	65	−63	66
**6c**	*FFE*	32	35	46	73	75	178	65	61	−65
**6d**	E*GE	55	−179	−59	180	−176	177	−68	64	61
**6e**	E*EE	60	−31	−46	−174	177	180	−64	65	65
**6f**	*EGE*	−59	177	−59	176	−176	178	64	63	62
**6g**	E*DE	48	28	−57	−179	−169	177	−61	53	69
**6h**	*DDD*	43	43	43	−173	−173	−173	49	49	49
**6i**	*EDE*	−46	56	−32	180	−172	−179	66	54	66
**6j**	*DFD*	41	34	47	−173	76	−177	50	63	60
**6k**	*DED*	27	−57	45	−169	177	−175	51	70	52
**7a**	*EEE*	−58	−58	−58	177	177	177	72	72	72
**7b**	EF*E	−56	−47	−54	178	−77	178	72	−52	72
**7c**	E*GE	58	180	−61	−179	−177	178	−70	69	67
**7d**	E*GF	59	−179	46	−179	−177	76	−72	70	52
**7e**	*DED*	37	−60	51	−172	175	−175	60	74	61
**7f**	E*EE	60	−38	−52	−174	179	180	−71	70	71
**7g**	*DFD*	52	48	56	−173	78	−174	60	51	65
**7h**	*DDD*	55	55	55	−173	−173	−173	59	59	59
**7i**	*EGE*	−56	178	−62	178	−176	178	70	68	69
**7j**	E*DE	53	38	−59	−179	−172	175	−70	61	73
**7k**	*EDE*	−60	38	−60	177	−172	175	73	61	74
**8a**	*EEE*	−57	−57	−57	178	178	178	72	72	72
**8b**	EF*E	−55	−45	−53	179	−76	180	72	−53	71
**8c**	E*GE	58	−179	−60	180	−177	178	−71	69	68
**8d**	E*GF	59	−178	46	180	−177	76	−72	71	53
**8e**	E*EE	60	−39	−52	−174	178	180	−71	70	70
**8f**	*DDD*	54	55	54	−173	−174	−174	58	58	58
**8g**	E*DE	53	39	−58	180	−171	176	−69	60	73
**8h**	*DFD*	52	48	55	−174	78	−174	60	51	63
**8i**	*EDE*	−59	41	−58	178	−171	176	72	60	73
**8j**	*EGE*	−55	179	−61	179	−176	179	70	68	69
**8k**	*DGD*	57	−179	52	−174	−178	−174	58	72	60
**8l**	*DED*	38	−59	51	−172	176	−175	59	73	61

^1^ Conformations of the substituents (the combination of dihedral angles κ, ν, and π; λ, ξ, and ρ; or µ, ο, and σ): **5** *D*—(*sc,-ap*,*sc*), *E*—(-*sc*,*ap*,*sc*), *F*—(*sc*,*sc*,*sc*), *G*—(*ap*,-*ap*,*sc*), *H*—(-*sc*,*sc*,*sc*); **6**–**8** *D*—(*sc*,-*ap*,*sc*), *E*—(-*sc*,*ap*,*sc*), *F*—(*sc*,*sc*,*sc*), *G*—(*ap*,-*ap*,*sc*); *sc*—*synclinal*, *ap—antiperiplanar*. * Mirror conformation.

## Data Availability

Data are contained within the article and Supplementary Materials.

## Supplementary Materials

The following supporting information can be downloaded at: https://www.mdpi.com/article/10.3390/molecules29010110/s1. Equations for α and γ calculations (Guggenheim–Smith equation); Figure S1: The ε*_i_*–*w_i_* and *n_i_*^2^–*w_i_* plots for compounds **1**–**4**, trichloromethane solutions; Figure S2: The ε*_i_*–*w_i_* and *n_i_*^2^–*w_i_* plots for compounds **5**, **7**, and **8**, trichloromethane solutions; Figure S3: The ε*_i_*–*w_i_* and *n_i_*^2^–*w_i_* plots for compounds **5** (1,4-dioxane solutions) and **8** (tetrachloromethane solutions); Table S1. Values of the slope (α or γ) and the intercept of the linear ε_i_–*w_i_* and *n_i_*^2^–*w_i_* dependences and standard errors of regression parameters for compounds **1**–**5**, **7**, **8**; Table S2. Selected vibration frequencies of **2**–**4**; theoretical values are listed for conformers **a**–**f**; Figure S4: FT-IR spectra of compound **3** in different aggregate states; Figure S5: FT-IR spectra of compound **4** in different aggregate states; Table S3. Selected vibration frequencies of **7**, **8**; theoretical values are listed for conformers 7**a**–**c**, **7f**, and **7h**–**j** and **8a**–**c**, **8e**–**g**, and **8j**.

## References

[1] Wallesch M., Volz D., Zink D.M., Schepers U., Nieger M., Baumann T., Bräse S. (2014). Bright coppertunities: Multinuclear CuI complexes with N–P ligands and their applications. Chem. Eur. J..

[2] Gneuss T., Leitl M.J., Finger L.H., Rau N., Yersin H., Sundermeyer J. (2015). A new class of luminescent Cu(I) complexes with tripodal ligands-TADF emitters for the yellow to red color range. Dalton Trans..

[3] Artem’ev A.V., Baranov A.Y., Rakhmanova M.I., Malysheva S.F., Samsonenko D.G. (2020). Copper(I) halide polymers derived from tris[2-(pyridin-2-yl)ethyl]phosphine: Halogen-tunable colorful luminescence spanning from deep blue to green. New J. Chem..

[4] Baranov A.Y., Berezin A.S., Samsonenko D.G., Mazur A.S., Tolstoy P.M., Plyusnin V.F., Kolesnikov I.E., Artem’ev A.V. (2020). New Cu(I) halide complexes showing TADF combined with room temperature phosphorescence: The balance tuned by halogens. Dalton Trans..

[5] Petrovskii S.K., Paderina A.V., Sizova A.A., Baranov A.Y., Artem’ev A.V., Sizov V.V., Grachova E.V. (2020). Luminescence behaviour of Au(I)–Cu(I) heterobimetallic coordination polymers based on alkynyl-tris(2-pyridyl)phosphine Au(I) complexes. Dalton Trans..

[6] Artem’ev A.V., Shafikov M.Z., Schinabeck A., Antonova O.V., Berezin A.S., Bagryanskaya I.Y., Plusnin P.E., Yersin H. (2019). Sky-blue thermally activated delayed fluorescence (TADF) based on Ag(I) complexes: Strong solvation-induced emission enhancement. Inorg. Chem. Front..

[7] Berezin A.S., Artem’ev A.V., Komarov V.Y., Baranov A.Y. (2020). A copper(I) bromide organic–inorganic zwitterionic coordination compound with a new type of core: Structure, luminescence properties, and DFT calculations. New J. Chem..

[8] Liu C.-Y., Chen X.-R., Chen H.-X., Niu Z., Hirao H., Braunstein P., Lang J.-P. (2020). Ultrafast luminescent light-up guest detection based on the lock of the host molecular vibration. J. Am. Chem. Soc..

[9] Petyuk M.Y., Berezin A.S., Gushchin A.L., Bagryanskaya I.Y., Baranov A.Y. (2021). Artem’ev, A.V. Re(I) scorpionates supported by tris(2-pyridyl)phosphine and its derivatives. Inorg. Chim. Acta.

[10] Baranov A.Y., Slavova S.O., Berezin A.S., Petrovskii S.K., Samsonenko D.G., Bagryanskaya I.Y., Fedin V.P., Grachova E.V., Artem’ev A.V. (2022). Controllable synthesis and luminescence behavior of tetrahedral Au@Cu_4_ and Au@Ag_4_ clusters supported by tris(2-pyridyl)phosphine. Inorg. Chem..

[11] Dayanova I.R., Fayezova A.I., Strelnik I.D., Litvinov I.A., Islamov D.R., Kolesnikov I.E., Gerasimova T.P., Musina E.I., Karasik A.A. (2022). Aurophilic interactions of dimeric bisphosphine gold(i) complexes pre-organized by the structure of the 1,5-diaza-3,7-diphosphacyclooctanes. Inorganics.

[12] Strelnik I.D., Dayanova I.R., Faizullin B.A., Mustafina A.R., Gerasimova T.P., Kolesnikov I.E., Islamov D.R., Litvinov I.A., Voloshina A.D., Sapunova A.S. (2023). Linkage of the dinuclear gold(i) complex luminescence and origin of endocyclic amino group of cyclic P2N2-bridging ligands. Inorg. Chem..

[13] Kharat A.N., Jahromi B.T., Bakhoda A. (2012). Manganese(II), cobalts(II) and nickel(II) complexes of tris(2-pyridyl)phosphine and their catalytic activity toward oxidation of tetralin. Transit. Met. Chem..

[14] Shuttleworth T.A., Miles-Hobbs A.M., Pringle P.G., Sparkes H.A. (2017). 2-Pyridyl substituents enhance the activity of palladium-phospha-adamantane catalysts for the methoxycarbonylation of phenylacetylene. Dalton Trans..

[15] Groutchik K., Jaiswal K., Dobrovetsky R. (2021). An air-stable, Zn^2+^-based catalyst for hydrosilylation of alkenes and alkynes. Org. Biomol. Chem..

[16] Wang X., Nurttila S.S., Dzik W.I., Becker R., Rodgers J., Reek J.N.H. (2017). Tuning the porphyrin building block in self-assembled cages for branched-selective hydroformylation of propene. Chem. Eur. J..

[17] Jacobs I., van Duin A.C.T., Kleij A.W., Kuil M., Tooke D.M., Spek A.L., Reek J.N.H. (2013). Conformational studies of ligand-template assemblies and the consequences for encapsulation of rhodium complexes and hydroformylation catalysis. Catal. Sci. Technol..

[18] Liu S., Pattacini R., Braunstein P. (2011). Reactions between an ethylene oligomerization chromium(III) precatalyst and aluminum-based activators: Alkyl and cationic complexes with a tridentate NPN ligand. Organometallics.

[19] Walden A.G., Miller A.J.M. (2015). Rapid water oxidation electrocatalysis by a ruthenium complex of the tripodal ligand tris(2-pyridyl)phosphine oxide. Chem. Sci..

[20] Huber W., Linder R., Niesel J., Schatzschneider U., Spingler B., Kunz P.C. (2012). A comparative study of tricarbonylmanganese photoactivatable CO releasing molecules (PhotoCORMs) by using the myoglobin assay and time-resolved IR spectroscopy. Eur. J. Inorg. Chem..

[21] Mansour A.M., Khaled R.M., Radacki K., Younes Z., Gamal M., Guirguis B., Mostafa G.A.E., Ali E.A., Shehab O.R. (2023). In vitro cytotoxicity of Mn(I) and Ru(II) carbonyls with a diphenyl pyridyl phosphine coligand towards leukaemia. Dalton Trans..

[22] Hu X.C., Sun T.Q., Zheng C.Y. (2018). Synthesis, crystal structures and magnetic properties of two iron (II) tris(pyridyl)phosphine selenides complexes. Phosphorus Sulfur Silicon.

[23] Zheng C., Hu X., Tao Q. (2018). Synthesis, structures and magnetic properties of two iron(ii) tris(pyridyl)phosphine sulfide complexes. Mendeleev Commun..

[24] You M., Nguyen G.T., Shao D., Wang T., Chang X., Ungur L., Zhang Y. (2022). Manipulating the spin crossover behaviour in a series of cyanide-bridged {Fe^III^_2_Fe^II^_2_} molecular squares through NCE^−^ co-ligands. Dalton Trans..

[25] García-Romero Á., Migue L.D., Wright D.S., Álvarez C.M., García-Rodríguez R. (2023). Structural and dimensional control of porphyrin capsules using Group 15 tris(3-pyridyl) linkers. Chem Sci..

[26] Jongkind L.J., Reek J.N.H. (2020). Asymmetric hydroformylation using a rhodium catalyst encapsulated in a chiral capsule. Chem. Asian J..

[27] Kharat A.N., Bakhoda A., Foroutannejad S., Foroutannejad C. (2011). Molecular structure and antimicrobial activity of a luminescent dinuclear silver(I) complex of phenyl-bis(2-pyridyl)phosphine. Z. Anorg. Allg. Chem..

[28] Artem’ev A.V., Eremina J.A., Liber E.V., Antonova O.V., Vorontsova E.V., Bagryanskaya I.Y. (2017). Luminescent Ag(I) scorpionates based on tris(2-pyridyl)phosphineoxide: Synthesis and cytotoxic activity evaluation. Polyhedron.

[29] Gülçin İ., Trofimov B., Kaya R., Taslimi P., Sobenina L., Schmidt E., Petrova O., Malysheva S., Gusarova N., Farzaliyev V. (2020). Synthesis of nitrogen, phosphorus, selenium and sulfur-containing heterocyclic compounds*—*Determination of their carbonic anhydrase, acetylcholinesterase, butyrylcholinesterase and α-glycosidase inhibition properties. Bioorg. Chem..

[30] Arbuzova S.N., Volkov P.A., Ivanova N.I., Gusarova N.K., Larina L.I., Kazheva O.N., Alexandrov G.G., Dyachenko O.A., Trofimov B.A. (2011). Synthesis and structural characterization of novel zinc(II) and cadmium(II) complexes with pyridine-phosphine chalcogenide ligands. J. Organomet. Chem..

[31] Plotnikova G.V., Malysheva S.F., Gusarova N.K., Khalliulin A.K., Udilov V.P., Kuznetsov K.L. (2008). Triorganylphosphine oxides as high-performance fire retardants for polyvinyl chloride plastisols. Russ. J. Appl. Chem..

[32] Hanf S., Colebatch A.L., Stehr P., García-Rodríguez R., Hey-Hawkins E., Wright D.S. (2020). An experimental and theoretical study of the coordination and donor properties of tris-2-pyridyl-phosphine ligands. Dalton Trans..

[33] Suter R., Sinclair H., Burford N., McDonald R., Ferguson M.J., Schrader E. (2017). Tris(2-pyridyl)phosphine as a versatile ligand for pnictogen acceptors. Dalton Trans..

[34] Whiteoak C.J., Nobbs J.D., Kiryushchenkov E., Pagano S., White A.J.P., Britovsek G.J.P. (2013). Tri(pyridylmethyl)phosphine: The elusive congener of TPA shows surprisingly different coordination behavior. Inorg. Chem..

[35] Artem’ev A.V., Kashevskii A.V., Bogomyakov A.S., Safronov A.Y., Sutyrina A.O., Telezhkin A.A., Sterkhova I.V. (2017). Variable coordination of tris(2-pyridyl)phosphine and its oxide toward M(hfac)_2_: A metal-specifiable switching between the formation of mono- and bis-scorpionate complexes. Dalton Trans..

[36] Baranov A.Y., Pritchina E.A., Berezin A.S., Samsonenko D.G., Fedin V.P., Belogorlova N.A., Gritsan N.P., Artem’ev A.V. (2021). Beyond classical coordination chemistry: The first case of a triply bridging phosphine ligand. Angew. Chem., Int. Ed..

[37] Dubován L., Pöllnitz A., Silvestru C. (2016). Tri(3-pyridyl)- and tri(4-pyridyl) phosphine chalcogenides and their complexes with ZnTPP (TPP = tetraphenylporphyrinate). Eur. J. Inorg. Chem..

[38] Keene F.R., Snow M.R., Tiekink E.R.T. (1988). Tris(2-pyridyl)phosphine. Acta Crystallogr. C Struct. Chem..

[39] Bowen R.J., Fernandes M.A., Gitari P.W., Layh M. (2004). Tris(2-pyridyl)phosphine oxide: How C-H^...^O and C-H^...^N interactions can affect crystal packing efficiency. Acta Crystallogr. C Struct. Chem..

[40] Kharat A.N., Bakhoda A., Hajiashrafi T., Abbasi A. (2010). Synthesis, characterization, and crystal structures of tris(2-pyridyl)phosphine sulfide and selenide. Phosphorus Sulfur Silicon.

[41] Vereshchagina Y.A., Chachkov D.V., Alimova A.Z., Malysheva S.F., Gusarova N.K., Ishmaeva E.A., Trofimov B.A. (2014). Dipole moments and conformational analysis of tris(2-pyridyl)phosphine and tris(2-pyridyl)phosphine chalcogenides. Experimental and theoretical study. J. Mol. Struct..

[42] Sterkhova I., Smirnov V., Artem’ev A., Trofimov B. (2014). CCDC 1031427: Experimental Crystal Structure Determination. CSD Commun..

[43] Hettstedt C., Unglert M., Mayer R.J., Frank A., Karaghiosoff K. (2016). Methoxyphenyl substituted bis(picolyl)phosphines and phosphine oxides. Eur. J. Inorg. Chem..

[44] Malysheva S.F., Belogorlova N.A., Kuimov V.A., Litvintsev Y.I., Sterkhova I.V., Albanov A.I., Gusarova N.K., Trofimov B.A. (2018). PCl_3_- and organometallic-free synthesis of tris(2-picolyl)phosphine oxide from elemental phosphorus and 2-(chloromethyl)pyridine hydrochloride. Tetrahedron Lett..

[45] Ustynyuk Y.A., Gloriozov I.P., Kalmykov S.N., Mitrofanov A.A., Babain V.A., Alyapyshev M.Y., Ustynyuk N.A. (2014). Pyridinedicarboxylic acid diamides as selective ligands for extraction and separation of trivalent lanthanides and actinides: DFT study. Solv. Extr. Ion Exch..

[46] Lavrov H.V., Ustynyuk N.A., Matveev P.I., Gloriozov I.P., Zhokhov S.S., Alyapyshev M.Y., Tkachenko L.I., Voronaev I.G., Babain V.A., Kalmykov S.N. (2017). A novel highly selective ligand for separation of actinides and lanthanides in the nuclear fuel cycle. Experimental verification of the theoretical prediction. Dalton Trans..

[47] Smirnova A., Yablonskiy M., Petrov V., Mitrofanov A. (2023). DFT prediction of radiolytic stability of conformationally flexible ligands. Energies.

[48] Mitrofanov A., Andreadi N., Matveev P., Zakirova G., Borisova N., Kalmykov S., Petrov V. (2021). An(III)/Ln(III) solvent extraction: Theoretical and experimental investigation of the role of ligand conformational mobility. J. Mol. Liq..

[49] Borisova N.E., Reshetova M.D. (2015). Quantum chemical modeling of 2,2′-bipyridine-6,6′-dicarboxylic acid diamide structures: A relationship between the extraction ability and conformational behavior of the ligands. Russ. Chem. Bull..

[50] Ongagna J.M., Fouegue A.D.T., Amana B.A., D’Ambassa G.M., Mfomo J.Z., Meva’A L.M., Mama D.B. (2020). B3LYP, M06 and B3PW91 DFT assignment of nd^8^metal-*bis*-(*N*-heterocyclic carbene) complexes. J. Mol. Model..

[51] Vo M.N., Bryantsev V.S., Johnson J.K., Keith J.A. (2018). Quantum chemistry benchmarking of binding and selectivity for lanthanide extractants. Int. J. Quantum Chem..

[52] Vereshchagina Y.A., Ismagilova R.R., Chachkov D.V., Malysheva S.F., Belogorlova N.A. (2019). Polarity and structure of Se-esters of diselenophosphinic acids: Experimental and theoretical conformational analysis in solution. Russ. J. Gen. Chem..

[53] Kuznetsova A.A., Chachkov D.V., Belogorlova N.A., Kuimov V.A., Malysheva S.F., Vereshchagina Y.A. (2021). Polarity and conformational analysis of tri(1-naphthyl)phosphine, tri(2-naphthyl)phosphine, and their chalcogenides. Russ. J. Org. Chem..

[54] Kuznetsova A.A., Chachkov D.V., Tcarkova K.V., Bondarenko N.A., Vereshchagina Y.A. (2021). Conformational analysis of dibutylphosphorylacetic acid *N*,*N*-dibutylamide in solution. Russ. J. Gen. Chem..

[55] Vereshchagina Y.A., Ishmaeva E.A., Zverev V.V. (2005). Theoretical conformational analysis of organophosphorus compounds. Russ. Chem. Rev..

[56] Ishmaeva E.A., Timosheva A.P., Timosheva N.V., Vereshchagina Y.A. (1998). Spravochnik po dipol’nym momentam fosfororganicheskih soedinenii. Handbook of Dipole Moments of Organophosphorus Compounds.

[57] Osipov O.A., Minkin V.I., Garnovskii A. (1971). Spravochnik po dipol’nym momentam. Handbook of Dipole Moments.

[58] Guggenheim E.A. (1949). A proposed simplification in the procedure for computing electric dipole moments. Trans. Faraday Soc..

[59] Smith J.W. (1950). Some developments of Guggenheim’s simplified procedure for computing electric dipole moments. Trans. Faraday Soc..

[60] Gribov L.A., Popov E.M. (1962). Valence-optical method and theoretical investigation of intensities and polarizations of fundamentals of polyatomic molecules in absorption spectra. Dokl. Akad. Nauk SSSR.

[61] Pimentel G.C., McClellan A.L. (1960). The Hydrogen Bond.

[62] Malysheva S., Kuimov V., Belovezhets L., Belogorlova N., Borovskaya M., Borovskii G. (2023). Phosphine chalcogenides and their derivatives from red phosphorus and functionalized pyridines, imidazoles, pyrazoles and their antimicrobial and cytostatic activity. Bioorg. Chem..

[63] Becke A.D. (1993). Density-functional thermochemistry. III. The role of exact exchange. J. Chem. Phys..

[64] Perdew J.P., Burke K., Wang Y. (1996). Generalized gradient approximation for the exchange-correlation hole of a many-electron system. Phys. Rev. B.

[65] McLean A.D., Chandler G.S. (1980). Contracted Gaussian basis sets for molecular calculations. I. Second row atoms, Z = 11–18. J. Chem. Phys..

[66] Frisch M.J., Trucks G.W., Schlegel H.B., Scuseria G.E., Robb M.A., Cheeseman J.R., Scalmani G., Barone V., Mennucci B., Petersson G.A. (2009). Gaussian 09.

[67] Medvedev M.G., Bushmarinov I.S., Sun J., Perdew J.P., Lyssenko K.A. (2017). Density functional theory is straying from the path toward the exact functional. Science.

[68] Cossi M., Rega N., Scalmani G., Barone V. (2003). Energies, structures, and electronic properties of molecules in solution with the C-PCM solvation model. J. Comp. Chem..

[69] Dennington R., Keith T.A., Millam J.M. (2016). GaussView, Version 6.

[70] (2023). Chemcraft (Version 1.7, Build 375). http://www.chemcraftprog.com.

[71] Zhang J., Zhang H., Wu T., Wang Q., van der Spoel D. (2017). Comparison of implicit and explicit solvent models for the calculation of solvation free energy in organic solvents. J. Chem. Theor. Comput..

[72] Vitkovskaya N.M., Orel V.B., Kobychev V.B., Bobkov A.S., Absalyamov D.Z., Trofimov B.A. (2020). Quantum-chemical models of KOH(KOBu^t^)/DMSO superbasic systems and mechanisms of base-promoted acetylene reactions. Int. J. Quantum Chem..

[73] Katsyuba S.A., Gerasimova T.P., Spicher S., Bohle F., Grimme S. (2022). Computer-aided simulation of infrared spectra of ethanol conformations in gas, liquid and in CCl_4_ solution. J. Comput. Chem..

