# Supplementary material for: Structure of Tris[2-(4-pyridyl)ethyl]phosphine, Tris[2-(2-pyridyl)ethyl]phosphine, and Their Chalcogenides in Solution: Dipole Moments, IR Spectroscopy, and DFT Study
Source: Molecules. 2023 Dec 23;29(1):110. doi: 10.3390/molecules29010110 (PMC10779502; doi:10.3390/molecules29010110)
Supplement: Supplementary file 1 [file molecules-29-00110-s001.zip › molecules-2744074-supplementary.pdf]

# Supplementary Materials

## Structure of Tris[2-(4-pyridyl)ethyl]phosphine, Tris[2-(2-pyridyl)ethyl]phosphine, and Their Chalcogenides in Solution: Dipole Moments, IR Spectroscopy, DFT Study

**Anastasiia A. Kuznetsova [1], Denis V. Chachkov [2], Natalia A. Belogorlova [3], Svetlana F. Malysheva [3] and Yana A. Vereshchagina [1,*]**

1 Department of Physical Chemistry, A.M. Butlerov Institute of Chemistry, Kazan Federal University, Kremlevskaya 18, 420008 Kazan, Russia; kuznetsovaanastan@gmail.com
2 Kazan Department of Joint Supercomputer Center of Russian Academy of Sciences–Branch of Federal Scientific Center "Scientific Research Institute for System Analysis of the RAS", Lobachevskogo 2/31, 420111 Kazan, Russia; de2005c@gmail.com
3 A.E. Favorsky Irkutsk Institute of Chemistry, Siberian Branch of the Russian Academy of Sciences, Favorskogo 1, 664033 Irkutsk, Russia; mal@irioch.irk.ru
* Correspondence: jveresch@kpfu.ru

### Contents

Equations for $\alpha$ and $\gamma$ calculations (Guggenheim-Smith equation) pS2
Figure S1. The $\varepsilon_i$–$w_i$ and $n_i^2$–$w_i$ plots for compounds **1–4**, trichloromethane solutions. pS3
Figure S2. The $\varepsilon_i$–$w_i$ and $n_i^2$–$w_i$ plots for compounds **5**, **7**, and **8**, trichloromethane solutions. pS4
Figure S3. The $\varepsilon_i$–$w_i$ and $n_i^2$–$w_i$ plots for compounds **5** (1,4-dioxane solutions) and **8** (tetrachloromethane solutions). pS5
Table S1. Values of the slope ($\alpha$ or $\gamma$) and the intercept of the linear $\varepsilon_i$–$w_i$ and $n_i^2$–$w_i$ dependences and standard errors of regression parameters for compounds **1–5**, **7**, **8**. pS6
Table S2. Selected vibration frequencies of **2–4**; theoretical values are listed for conformers **a–f**. pS7
Figure S4. FT-IR spectra of compound **3** in different aggregate states. pS8
Figure S5. FT-IR spectra of compound **4** in different aggregate states. pS9
Table S3. Selected vibration frequencies of **7**, **8**; theoretical values are listed for conformers 7**a–c**, **7f**, **7h-j** and **8a–c**, **8e-g**, **8j**. pS10

Equations for $\alpha$ and $\gamma$ calculations (Guggenheim-Smith equation):

$$\alpha = \frac{\varepsilon_i - \varepsilon_0}{w_i},$$

$$\gamma = \frac{n_i^2 - n_0^2}{w_i},$$

where $\alpha$ and $\gamma$ are slopes of the $\varepsilon_i$–$w_i$ and $n_i^2$–$w_i$ plots; $\varepsilon_i$, $n_i$, and $w_i$ are the dielectric permittivity, refractive index, and weigh fraction of the solute of the $i^{th}$ solution, respectively.

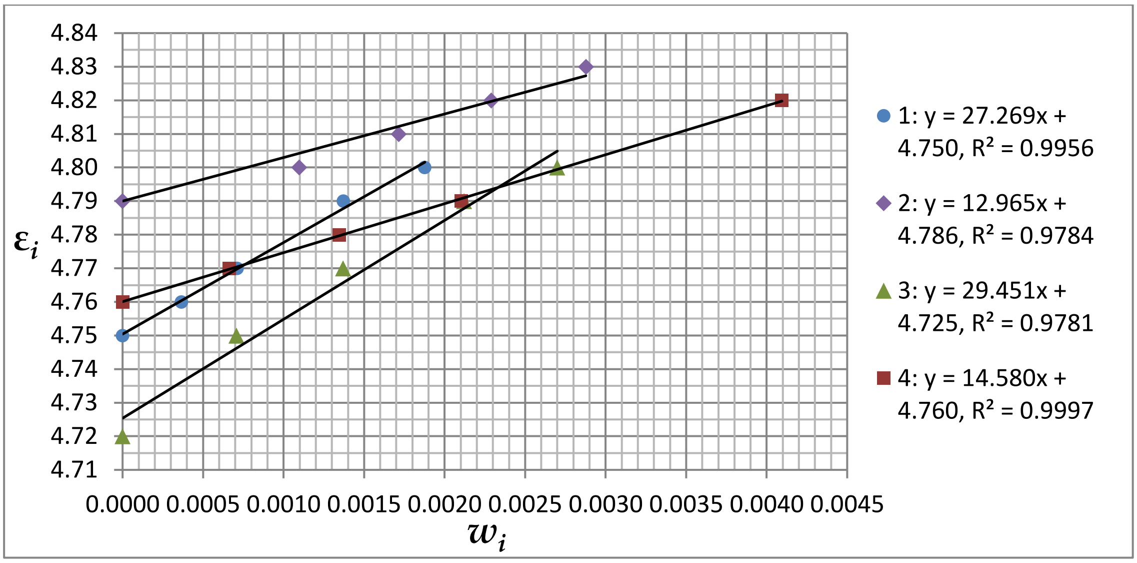


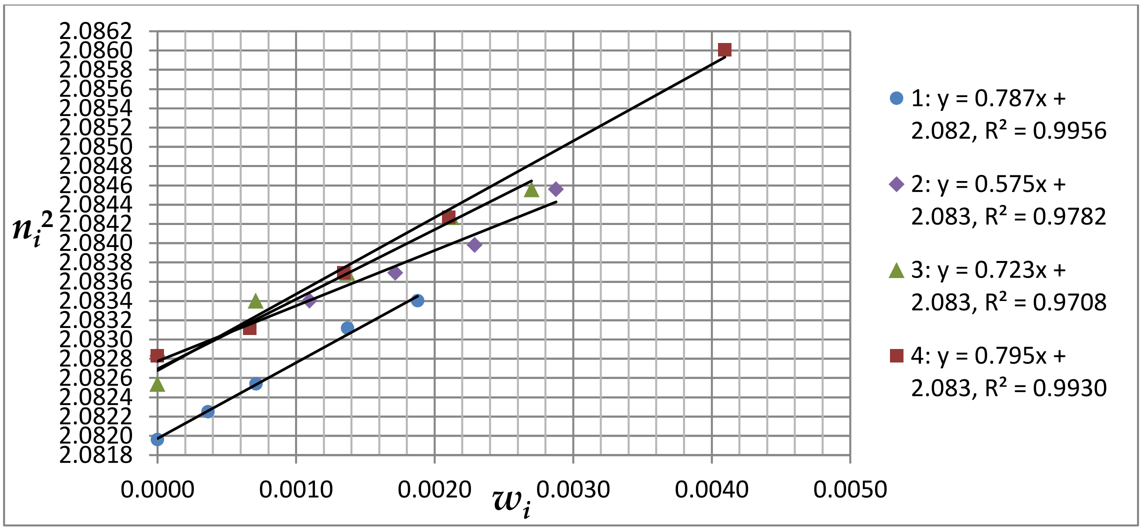


**Figure S1.** The $\varepsilon_i$–$w_i$ and $n_i^2$–$w_i$ plots for compounds **1-4**, trichloromethane solutions.

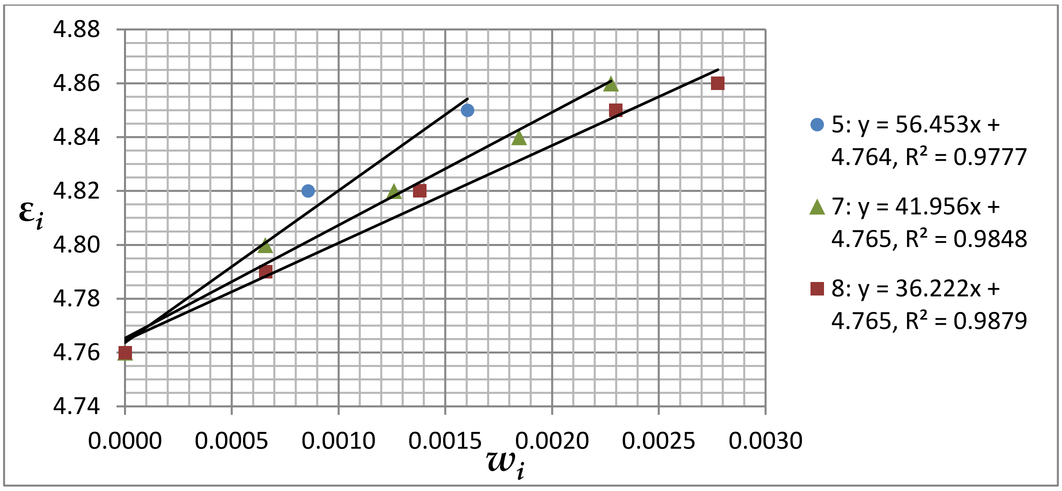


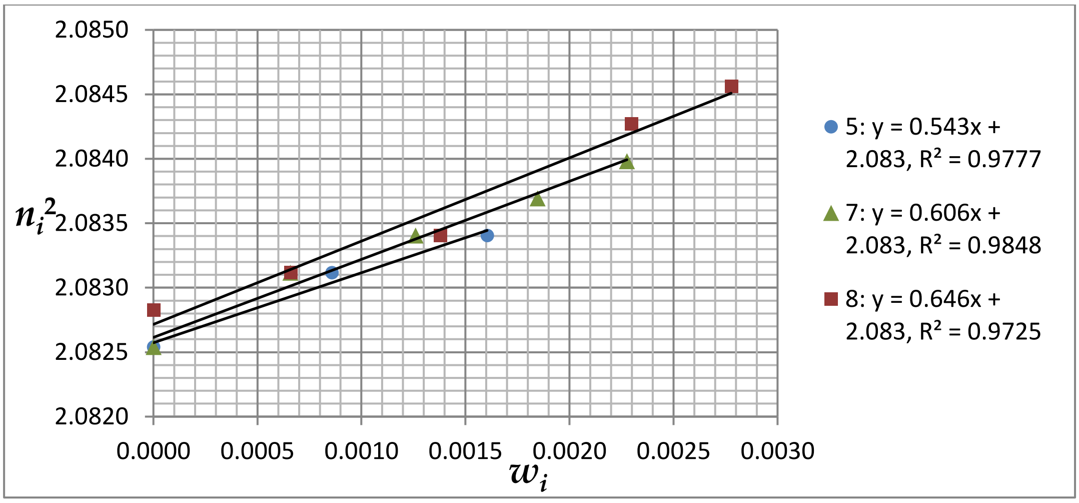


**Figure S2.** The $\varepsilon_i$–$w_i$ and $n_i^2$–$w_i$ plots for compounds **5**, **7**, and **8**, trichloromethane solutions.

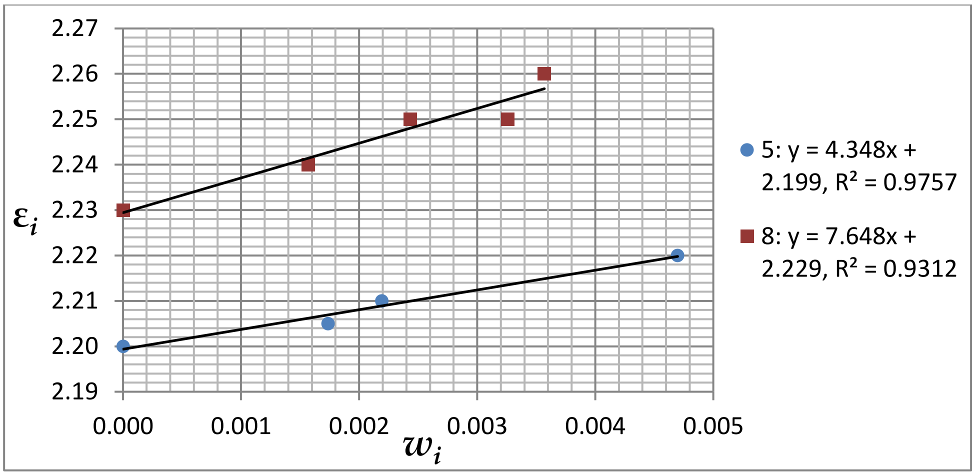


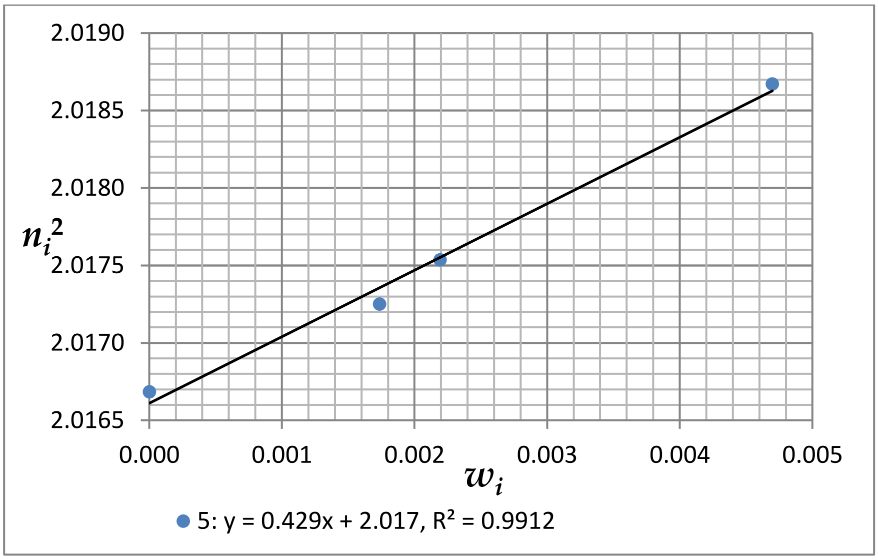


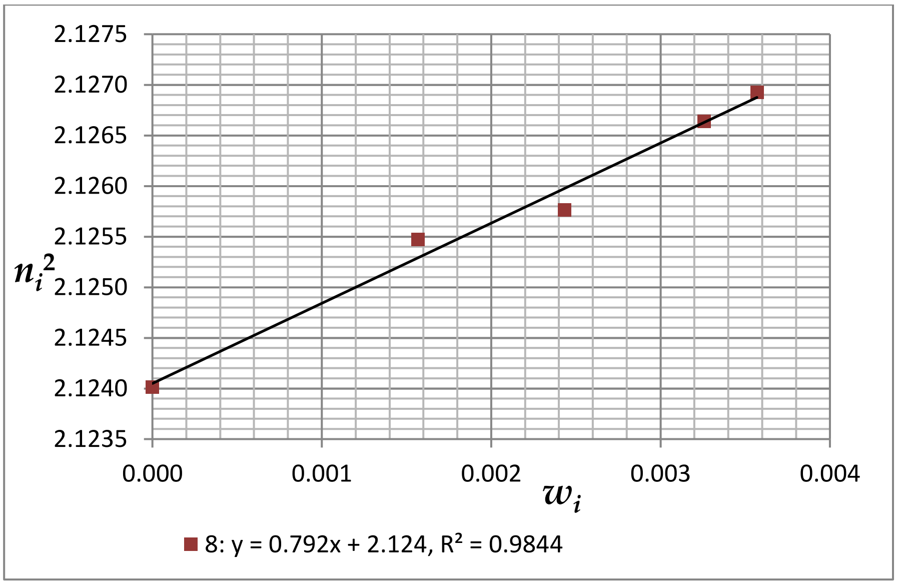


**Figure S3.** The $\varepsilon_i$–$w_i$ and $n_i^2$–$w_i$ plots for compounds **5** (1,4-dioxane solutions) and **8** (tetrachloromethane solutions).

**Table S1.** Values of the slope ($\alpha$ or $\gamma$) and the intercept of the linear $\varepsilon_i$–$w_i$ and $n_i^2$–$w_i$ dependences and standard errors of regression parameters for compounds **1–5**, **7**, **8**.

| | Solvent | $\varepsilon_i = \alpha \cdot w_i + c_1$ | | | | $n_i^2 = \gamma \cdot w_i + c_2$ | | | |
|---|---|---|---|---|---|---|---|---|---|
| | | $\alpha$ | $\Delta\alpha$ | $c_1$ | $\Delta c_1$ | $\gamma$ | $\Delta\gamma$ | $c_2$ | $\Delta c_2$ |
| **1** | Trichloromethane | 27.27 | 1.05 | 4.76 | 0.00 | 0.787 | 0.030 | 2.0820 | 0.0000 |
| **2** | Trichloromethane | 12.97 | 1.10 | 4.79 | 0.00 | 0.575 | 0.050 | 2.0828 | 0.0001 |
| **3** | Trichloromethane | 29.45 | 2.54 | 4.73 | 0.00 | 0.723 | 0.072 | 2.0827 | 0.0001 |
| **4** | Trichloromethane | 14.58 | 0.16 | 4.76 | 0.00 | 0.795 | 0.038 | 2.0827 | 0.0001 |
| **5** | Trichloromethane | 56.45 | 8.52 | 4.76 | 0.01 | 0.543 | 0.082 | 2.0826 | 0.0001 |
| | 1,4-Dioxane | 4.35 | 0.49 | 2.20 | 0.00 | 0.429 | 0.029 | 2.0166 | 0.0001 |
| **7** | Trichloromethane | 41.96 | 3.01 | 4.77 | 0.00 | 0.606 | 0.043 | 2.0826 | 0.0001 |
| **8** | Trichloromethane | 36.22 | 2.32 | 4.76 | 0.00 | 0.646 | 0.063 | 2.0827 | 0.0001 |
| | Tetrachloromethane | 7.65 | 1.20 | 2.23 | 0.00 | 0.792 | 0.057 | 2.1241 | 0.0001 |

**Table S2.** Selected vibration frequencies ($cm^{-1}$) of **2–4**; theoretical values are listed for conformers **a–f**.

| **Vibrations*** | **Experimental** | | | **Gas phase** | | | | | | **Solution (CPCM model)** | | | | | |
|---|---|---|---|---|---|---|---|---|---|---|---|---|---|---|---|
| | **Solid** | **Solution** | **Melt** | **2a** | **2b** | **2c** | **2d** | **2e** | **2f** | **2a** | **2b** | **2c** | **2d** | **2e** | **2f** |
| C–H(Pyridyl) (ω) | 805 | - | 802 | 822 | 819, 825 | 817, 828 | 822 | 821 | 819 | 823 | 822 | 817, 825 | 823 | 823 | 820 |
| C–H(Ethyl) (τ, ϱ) | 953, 975 | 925, 939 | 948 | 948 | 925, 949 | 911, 929, 953 | 919, 948, 969 | 927, 945, 969 | 958 | 950 | 923, 952 | 902, 923, 956 | 926, 953, 970 | 930, 950, 971 | 959 |
| C–H(Pyridyl) (τ) | 994 | 995 | 991 | 1013 | 1014 | 1015 | 1016 | 1012 | 1017 | 1015 | 1014 | 1015 | 1015 | 1015 | 1015 |
| P=O (ν) | 1149, 1168 | 1167 | 1169 | 1211 | 1206 | 1204 | 1211 | 1196 | 1197 | 1181 | 1183 | 1283 | 1183 | 1172 | 1172 |
| C–H(Pyridyl) (δ) | 1415 | 1418 | 1414 | 1449 | 1449 | 1447 | 1449 | 1449 | 1447 | 1447 | 1446 | 1447 | 1446 | 1446 | 1446 |
| C=C and C=N (ν) | 1599 | 1603 | 1597 | 1647 | 1648 | 1648 | 1647 | 1646 | 1648 | 1648 | 1647 | 1648 | 1647 | 1647 | 1647 |
| | | | | **3a** | **3b** | **3c** | **3d** | **3e** | **3f** | **3a** | **3b** | **3c** | **3d** | **3e** | **3f** |
| C–H(Pyridyl) (ω) | 799, 807 | no | 802 | 821 | 822 | 816, 822 | 822 | 820 | 821 | 823 | 824 | 816, 822 | 822 | 822 | 823 |
| C–H(Ethyl) (τ, ϱ) | 939, 956, 967 | 939, 953 | 952 | 945, 971 | 902, 923, 949, 964 | 905, 920, 932, 959 | 922, 949, 984 | 920, 964 | 939, 948, 972 | 950, 971 | 901, 924, 951, 963 | 902, 922, 934, 961 | 932, 949, 982 | 923, 964, 973 | 939, 950, 973 |
| C–H(Pyridyl) (τ) | 994 | 995 | 991 | 1015 | 1015 | 1015 | 1014 | 1014 | 1013 | 1015 | 1013 | 1014 | 1014 | 1014 | 1015 |
| C–H(Pyridyl) (δ) | 1418 | 1417 | 1414 | 1 448 | 1447 | 1447 | 1447 | 1448 | 1449 | 1446 | 1448 | 1447 | 1446 | 1449 | 1446 |
| C=C and C=N (ν) | 1602 | 1603 | 1597 | 1648 | 1648 | 1648 | 1648 | 1648 | 1647 | 1647 | 1647 | 1648 | 1647 | 1647 | 1647 |
| | | | | **4a** | **4b** | **4c** | **4d** | **4e** | **4f** | **4a** | **4b** | **4c** | **4d** | **4e** | **4f** |
| C–H(Pyridyl) (ω) | 807 | no | | 822 | 822 | 815, 823 | 822 | 818 | 822 | 824 | 816, 822 | 815, 821 | 822 | 820 | 824 |
| C–H(Ethyl) (τ, ϱ) | 938, 956, 967 | 924, 939, 952, | | 946, 967 | 902, 922, 949, 961 | 903, 921, 933, 960 | 923, 946, 982 | 963, 968 | 937, 948, 972 | 951, 963 | 902, 922, 952, 964 | 901, 921, 933, 963 | 925, 952, 976 | 962, 974 | 939, 951, 974 |
| C–H(Pyridyl) (τ) | 995 | 995 | | 1014 | 1014 | 1016 | 1014 | 1016 | 1013 | 1013 | 1014 | 1013 | 1014 | 1015 | 1016 |
| C–H(Pyridyl) (δ) | 1419 | 1418 | | 1450 | 1449 | 1448 | 1448 | 1449 | 1449 | 1 447 | 1446 | 1445 | 1446 | 1446 | 1447 |
| C=C and C=N (ν) | 1602 | 1603 | | 1647 | 1647 | 1649 | 1648 | 1647 | 1647 | 1648 | 1647 | 1647 | 1647 | 1647 | 1647 |

* ν – stretching, δ – scissoring, ϱ – rocking, ω – wagging, τ – twisting

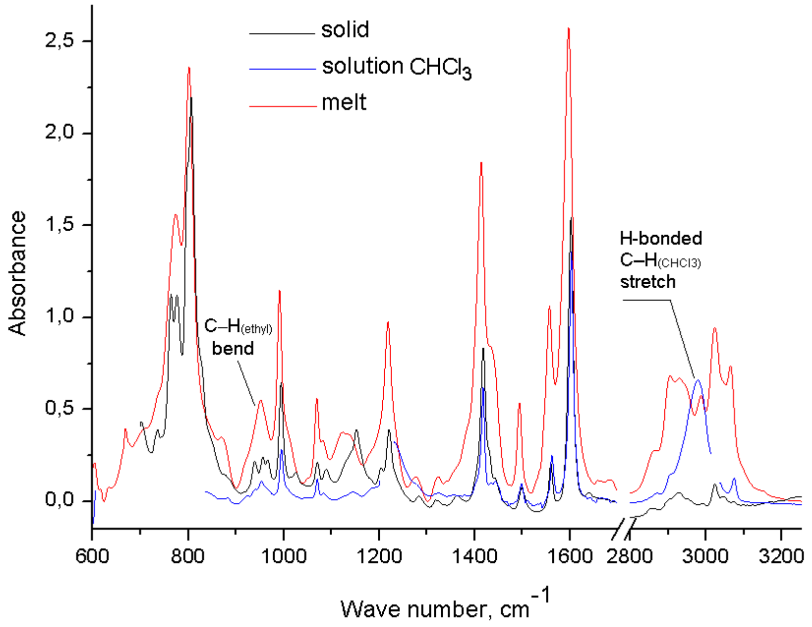


**Figure S4.** FT-IR spectra of compound **3** in different aggregate states.

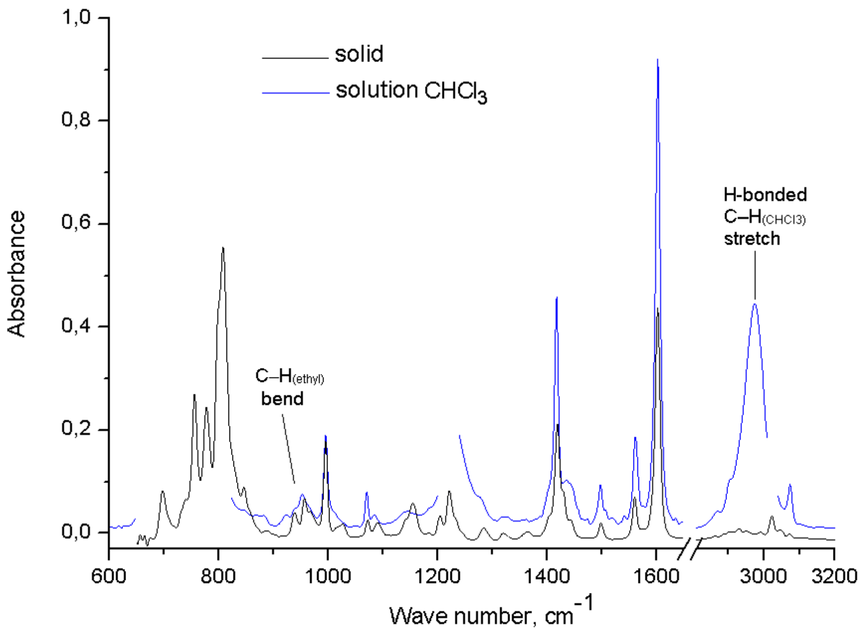


**Figure S5.** FT-IR spectra of compound **4** in different aggregate states.

**Table S3.** Selected vibration frequencies ($cm^{-1}$) of **7**, **8**; theoretical values are listed for conformers 7**a–c**, **7f**, **7h-j** and **8a–c**, **8e-g**, **8j**.

| Vibrations* | Experimental | | Gas phase | | | | | | | Solution (CPCM model) | | | | | | |
|---|---|---|---|---|---|---|---|---|---|---|---|---|---|---|---|---|
| | Solid | Solution | 7a | 7b | 7c | 7f | 7h | 7i | 7j | 7a | 7b | 7c | 7f | 7h | 7i | 7j |
| C–$H_{(Pyridyl)}$ (ω) | 798, 808 | no | 795 | 788, 797 | 778, 799 | 796, 801 | 794 | 790, 799 | 788, 797 | 798 | 785, 799 | 777, 797 | 799 | 789, 798 | 798 | 793, 799 |
| C–$H_{(Ethyl)}$ (τ, ϱ) | 940, 957, 968 | 939, 952, 965 | 950, 970 | 919, 930, 955, 966 | 968 | 953, 977, 989 | 951, 966 | 945, 974 | 953, 978, 990 | 950, 971 | 930, 954, 966 | 964, 973 | 953, 977 | 953, 970 | 943, 952, 973 | 953, 976 |
| C–$H_{(Pyridyl)}$ (τ) | 995 | 997 | 1012 | 1014 | 1012 | 1012 | 1013 | 1013 | 1013 | 1012 | 1013 | 1014 | 1013 | 1012 | 1012 | 1013 |
| C–$H_{(Pyridyl)}$ (δ) | 1419 | 1436 | 1466, 1478 | 1465, 1474 | 1466, 1476 | 1467, 1476 | 1470 | 1467, 1476 | 1467, 1476 | 1464, 1473 | 1471 | 1472 | 1465, 1474 | 1463, 1472 | 1463, 1472 | 1465, 1470 |
| C–$H_{(Pyridyl)}$ (ϱ) | - | 1475 | 1510 | 1510 | 1508 | 1509 | 1509 | 1509 | 1509 | 1508 | 1510 | 1508 | 1509 | 1508 | 1508 | 1509 |
| C=C and C=N (ν) | 1602 | 1593 | 1644 | 1643 | 1642 | 1642 | 1642 | 1642 | 1642 | 1642 | 1641 | 1641 | 1640 | 1642 | 1642 | 1640 |
| | | | 8a | 8b | 8c | 8e | 8f | 8g | 8j | 8a | 8b | 8c | 8e | 8f | 8g | 8j |
| C–$H_{(Pyridyl)}$ (ω) | 750, 766 | - | 765, 795 | 764, 785, 797 | 766, 775, 796 | 765, 795 | 761, 788, 796 | 764, 773, 788, 797 | 764, 773, 796 | 766, 799 | 764, 785, 797 | 765, 777, 798 | 767, 799 | 758, 767, 787, 799 | 768, 792 | 770, 797 |
| C–$H_{(Ethyl)}$ (τ, ϱ) | 938, 948, 966 | 922, 939, 951, 965 | 949, 967 | 931, 954, 966 | 965 | 953, 976, 988 | 951, 963 | 954, 978, 988 | 945, 954, 974 | 950, 965 | 955, 967 | 964, 973 | 954, 978 | 951, 968 | 955, 976 | 943, 952, 975 |
| C–$H_{(Pyridyl)}$ (τ) | 994 | 997 | 1015 | 1014 | 1013 | 1012 | 1013 | 1013 | 1013 | 1013 | 1014 | 1012 | 1013 | 1013 | 1014 | 1014 |
| C–$H_{(Pyridyl)}$ (δ) | 1435 | 1437 | 1465, 1477 | 1465, 1474 | 1467, 1476 | 1466, 1475 | 1470 | 1467, 1476 | 1467, 1476 | 1464, 1473 | 1463, 1472 | 1466, 1472 | 1465, 1474 | 1470 | 1463, 1472 | 1466, 1472 |
| C–$H_{(Pyridyl)}$ (ϱ) | 1475 | 1475 | 1510 | 1509 | 1508 | 1508 | 1509 | 1509 | 1509 | 1509 | 1507 | 1508 | 1507 | 1509 | 1508 | 1507 |
| C=C and C=N (ν) | 1589 | 1594 | 1643 | 1643 | 1642 | 1641 | 1642 | 1642 | 1642 | 1639 | 1641 | 1642 | 1640 | 1640 | 1641 | 1641 |

* ν – stretching, δ – scissoring, ϱ – rocking, ω – wagging, τ – twisting
